# Challenging Structure Elucidation of Lumnitzeralactone, an Ellagic Acid Derivative from the Mangrove *Lumnitzera racemosa*

**DOI:** 10.3390/md21040242

**Published:** 2023-04-14

**Authors:** Jonas Kappen, Jeprianto Manurung, Tristan Fuchs, Sahithya Phani Babu Vemulapalli, Lea M. Schmitz, Andrej Frolov, Andria Agusta, Alexandra N. Muellner-Riehl, Christian Griesinger, Katrin Franke, Ludger A. Wessjohann

**Affiliations:** 1 Department of Bioorganic Chemistry, Leibniz Institute of Plant Biochemistry (IPB), Weinberg 3, 06120 Halle (Saale), Germany; jkappen@ipb-halle.de (J.K.); jeprianto_m@apps.ipb.ac.id (J.M.); tfuchs@ipb-halle.de (T.F.); lschmitz@ipb-halle.de (L.M.S.); andrej.frolov@ipb-halle.de (A.F.); 2 Department of Molecular Evolution and Plant Systematics & Herbarium (LZ), Institute of Biology, Leipzig University, Johannisallee 21-23, 04103 Leipzig, Germany; muellner-riehl@uni-leipzig.de; 3 German Centre for Integrative Biodiversity Research (iDiv) Halle-Jena-Leipzig, Puschstraße 4, 04103 Leipzig, Germany; 4 Department of NMR-Based Structural Biology, Max Planck Institute for Multidisciplinary Sciences, Am Fassberg 11, 37077 Göttingen, Germany; save@mpinat.mpg.de; 5 Research Group for Marine Geochemistry, Institute for Chemistry and Biology of the Marine Environment (ICBM), Carl von Ossietzky Universität Oldenburg, Carl-von-Ossietzky-Str. 9-11, 26129 Oldenburg, Germany; 6 Research Center for Pharmaceutical Ingredients and Traditional Medicine, National Research and Innovation Agency (BRIN), Jl. M.H. Thamrin No. 8, Jakarta 10340, Indonesia; andr005@brin.go.id; 7 Institute of Biology/Geobotany and Botanical Garden, Martin Luther University Halle-Wittenberg, 06108 Halle (Saale), Germany

**Keywords:** *Lumnitzera racemosa*, lumnitzeralactone, isolation, synthesis, structure elucidation, ellagic acid, anti-bacterial, NMR, ^13^C-^1^H ADEQUATE

## Abstract

The previously undescribed natural product lumnitzeralactone (**1**), which represents a derivative of ellagic acid, was isolated from the anti-bacterial extract of the Indonesian mangrove species *Lumnitzera racemosa* Willd. The structure of lumnitzeralactone (**1**), a proton-deficient and highly challenging condensed aromatic ring system, was unambiguously elucidated by extensive spectroscopic analyses involving high-resolution mass spectrometry (HRMS), 1D ^1^H and ^13^C nuclear magnetic resonance spectroscopy (NMR), and 2D NMR (including 1,1-ADEQUATE and 1,n-ADEQUATE). Determination of the structure was supported by computer-assisted structure elucidation (CASE system applying ACD-SE), density functional theory (DFT) calculations, and a two-step chemical synthesis. Possible biosynthetic pathways involving mangrove-associated fungi have been suggested.

## 1. Introduction

Mangroves are salt-tolerant plants, growing as shrubs or trees along coastlines at tropical and subtropical latitudes [1,2]. Together with associated microbes, fungi, other plants, and animals, they form a mangrove forest community also called mangal [2]. Altogether, about 75 true mangrove species from 11 families are recognized [3,4,5]. *Lumnitzera racemosa* Willd. belongs to the mostly (sub)tropical family Combretaceae. The species is widely distributed from the shores of East Africa to the Indo-West Pacific [6], as well as in the Malay Archipelago [2]. Its extracts are well known in traditional medicine, being used, among other applications, for the treatment of snake bites, rheumatism, skin allergies, asthma, and diabetes mellitus and as a blood purifier [2,7,8,9,10]. The fruits and juice of young twigs, as well as sap of older bark, are used for treating skin disorders, herpes, pruritus, and thrush arising from fungal infections [6,11,12,13]. Biological activities of *L. racemosa* extracts and constituents were intensively studied. Anti-bacterial [6,14,15,16], anti-coagulant [17], anti-inflammatory [18], anti-cancer [17,19,20], anti-angiogenic [18], anti-oxidative [17,20], hepatoprotective [21,22], anti-hypertensive [23], anti-hyperglycemic [24], and anti-viral effects [16,25,26,27] have been described so far. The reported major classes of secondary metabolites present in *L. racemosa* extracts comprise tannins [17,20,23], flavonoids [10,17,18,20,22], phenols [17,18,20,21], alkaloids [21], and terpenes [6,17,20,21]. Gallic acid and its derivatives—soluble tannins and related compounds such as ellagic acid (EA) or 3,3′,4-tri-*O*-methylellagic acid (TMEA)—were found as one of the possible biologically active classes of compounds [1,6,8,18,22]. These compounds are common for the Combretaceae family in general and for *L. racemosa* in particular. Recently, triterpene acids were identified as anti-bacterial compounds in Combretaceae [28], and their effect against *Staphyllococcus aureus* was proposed to be a result of synergism with epicatechin [28]. However, not all bioactive compounds of mangroves originate from the plant itself, with many produced by associated microorganisms [6,29,30,31], including various fungal endophytes. While the same fungal species have been found on different host plant species, multiple samples of the same mangrove species do not necessarily bear the same microorganisms [32,33]. It is likely that differences in the diversity of associated microorganisms are due to species composition and environmental conditions, such as precipitation, frequency of tidal flooding, salinity, freshwater flow, soil types, and hours of radiation at the place of occurrence, as cases of no strict host specificity are known [32].

Recently, in our comprehensive metabolomics survey, we characterized the patterns of ellagic acid derivatives in the root extracts of *L. racemosa* [8]. In the present study, we report the isolation, structure elucidation, and chemical synthesis of the previously undescribed ellagic acid derivative lumnitzeralactone (**1**) from *L. racemosa*. In addition, anti-bacterial activity was studied and putative biosynthetic pathways involving fungal participation are discussed.

## 2. Results and Discussion

### 2.1. Isolation and Identification of Compound **1**

**Figure 1:**
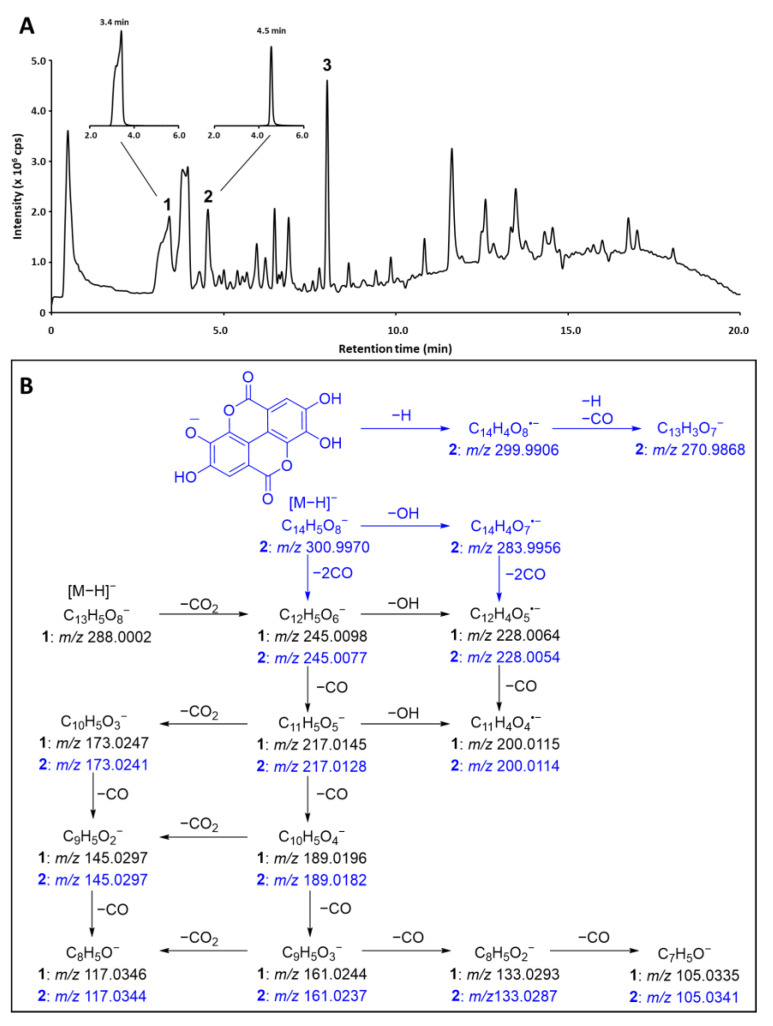
(**A**) Total ion chromatogram (TIC) acquired for the anti-bacterial *L. racemosa* sample LR7 along with the corresponding extracted ion chromatograms of *m/z* 289.0 ± 0.5 (**1**) and 301.0 ± 0.5 (**2**). Peak 3 represents 3,3′,4′-tri-*O*-methylellagic acid (**3**). (**B**) Suggested tandem mass spectrometric fragmentation (MS/MS) patterns of **1** and **2.** Fragments specific for EA are marked blue and fragments common for both compounds are in black.

**Figure 2:**
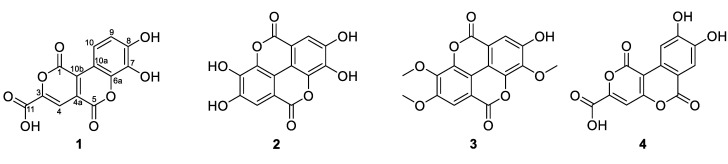
Structures of lumnitzeralactone (**1**), ellagic acid (EA, **2**), 3,3′,4′-tri-*O*-methylellagic acid (TMEA, **3**), and phelligridin J (**4**).

During our previous LC-MS investigation of 31 extracts from air-dried root samples of the Indonesian mangroves *L. racemosa* and *L. littorea*, a series of interesting new sulfated natural products as well as unusual EA derivatives were identified and subsequently isolated [8]. The extracts from mangroves from different locations varied significantly in their anti-bacterial activity. Remarkably, only two extracts obtained from locations close to each other (the Maluku islands Ternate and Halmahera) completely inhibited the growth of the Gram-positive bacterium *Bacillus subtilis* when applied at 500 µg/mL [8,34], which could indicate a connection between the locations of the plants and their anti-bacterial activity. This activity correlated with the occurrence of a signal at *m/z* 289 [M − H]^−^ (peak 1 in the chromatogram in Figure 1A) in the LC-MS profiles, which could be exclusively observed in the two active extracts. High-resolution mass spectrometry (HRMS) indicated the molecular formula C_13_H_6_O_8_ for **1**, based on the [M − H]^−^ ion at *m/z* 289.0002 calculated for C_13_H_5_O_8_^−^ to be 288.9990 with a mass tolerance of 4.2 ppm (Figure S5). Thus, as can be seen from the mass difference of 12 amu, **1** has one carbon atom less than EA (**2**). This elemental composition corresponds to only one known natural product, phelligridin J (**4**) (3-carboxyl-8,9-dihydroxypyrano[4,3-c]isochromen-4-one) (Figure 2), isolated from the Chinese medicinal fungus *Phellinus igniarius* [35]. However, the MS^2^ investigations of **1** revealed a fragmentation behavior similar to **2**, suggesting a close structural relationship between these two compounds (Figure 1B). Indeed, the fragmentation of **1** and **2** followed the pathways characteristic for phenolic compounds, as was recently described by Schmidt [36]. These pathways, accompanied with multiple losses of CO and CO_2_ and the formation of odd-electron *O*-centered radical ion intermediates, provide a good explanation for the observed patterns.

EA (**2**) is a ubiquitous secondary metabolite in plants particularly found in the Combretaceae family and quite characteristic of the genus *Lumnitzera*. The difference of 12 amu, observed between **1** and **2**, implies the potential occurrence of either a five-membered ring in **1,** instead of a six-membered ring in **2**, or a rearranged structure with the formal elimination of a (quarternary) carbon atom. To allow for unequivocal structure elucidation by NMR data, the compound had to be isolated.

An initial isolation approach under non-acidic conditions yielded 2.5 mg of **1,** which was only slightly soluble in methanol. Although initial preliminary NMR data could be obtained (Table S1), the amount was insufficient for further structure elucidation by 2D NMR. From the small amount of the remaining dried roots (15.06 g), an additional 14.6 mg of **1** was obtained. Acidification of the aqueous phase during liquid–liquid extraction of the crude extract with ethyl acetate allowed for extraction of the yellow-colored compound into the organic phase. Subsequently, **1** was purified by repeated column chromatography on Sephadex LH20 and reversed-phase 2 material (RP2), followed by semi-preparative RP-HPLC.

### 2.2. Structure Elucidation

Compound **1** was obtained as a yellow amorphous solid. Over time and through repeated dissolving and drying, the substance turned red and became less soluble in methanol. The bathochromic shift was reversible, and the color change could be repeated on a TLC plate (Figure S1). Under NH_3_ vapor, the color of the yellow spot of **1** immediately changed to red and then back to bright yellow when treated with HCl vapor. By spraying the plate with a solution of magnesium acetate in methanol, the red color could be fixed due to the formation of the corresponding phenolate ions (Mechanism: Scheme S1) [37]. This effect is known as the Bornträger reaction. The decreased solubility in methanol, also observed during the first extraction under neutral conditions, might result from stable salts formed by the phenolate ions, which strongly enhances the polarity of the molecule. The observed halochromism, namely the color change through salt formation by charge change of a molecule, is a result of an extensive electron delocalization due to the participation of the free electrons of the negative-charged oxygen of the phenolate. This is a strong hint for the presence of phenolic hydroxyl groups in **1**, which was not unexpected for an EA derivative. In agreement with data from the literature [37,38,39], the presence of a benzene ring, conjugated carbonyl groups, and phenolic hydroxyl groups can be assumed based on the presence of the maxima at 232 (4.10), 290 (3.95), and 407 (3.96) nm in the UV-Vis absorption spectra of **1** (Figure S9-1).

The ^1^H NMR spectrum of **1**, recorded in CD_3_OD, revealed three proton signals at δ_H_ 6.87 (d, 9 Hz), 7.59 (s), and 8.46 (d, 9 Hz) (Figure S2-1, Table S1-1), two of which (δ_H_ 6.87 and 8.46) are *ortho*-coupled, which was supported by the correlation observed in the COSY and TOCSY spectra (Figures S2-2 and S2-3). This is not compatible with a structure similar to **2**, which would show only two aromatic singlets. Usually, the coupling constant for protons of the benzene ring in the *ortho* position is in the range of 7.6–8.5 Hz [40,41]. Nevertheless, larger coupling constants are known. For example, urolithin M5, a degradation product of **2** detected in extracts from *Elaeocarpus tonkinensis* and also formed in humans after ingestion of **2** [42], showed a coupling constant of 9 Hz, which was the same as what was observed in **1**.

The ^1^H NMR spectrum of **1,** recorded in DMSO-*d6* (Table 1, Figure S2-7), shows two additional signals attributable to phenolic hydroxyl protons (δ_H_ 9.56, brs, 10.62, brs). The ^13^C NMR spectrum of **1,** recorded in DMSO-*d6* (Table 1, Figure S2-8), revealed 13 carbon signals, which is in agreement with the molecular formula. Three carboxyl or lactone carbon signals at δ_C_ 160.0, 158.3, and 158.2 were visible, as well as ten more carbons, seven of which were non-protonated, including two oxygen-bearing carbons at δ_C_ 132.7 and 150.8. The three protonated sp^2^ carbons were assigned by HSQC for δ_C_ 113.2, 117.9, and 107.3. Surprisingly, the ^13^C NMR (CD_3_OD) of **1**, isolated under non-acidic conditions, showed only 11 of the expected 13 carbon signals (Figure S2-4, Table S1-2). Nevertheless, the two missing signals (C-3 and C-11) could be determined by HMBC correlations (Figure S2-6). The higher chemical shift values of these signals indicated the presence of a salt instead of the free acid.

Based on the acquired data set, it seemed highly probable that the structure of **1** contains three protonated sp2 carbons (two of which are *ortho*-coupled aromatic protons), one carboxyl group, and two phenolic hydroxyl groups. Thus, a condensed system of three rings, including two lactones, was most likely, i.e., a structure representing a regioisomer of phelligridin J (**4**) [35]. Since **1** contains only few protons, COSY and HMBC correlations were not sufficient to elucidate its complete structure.

**Figure 3:**
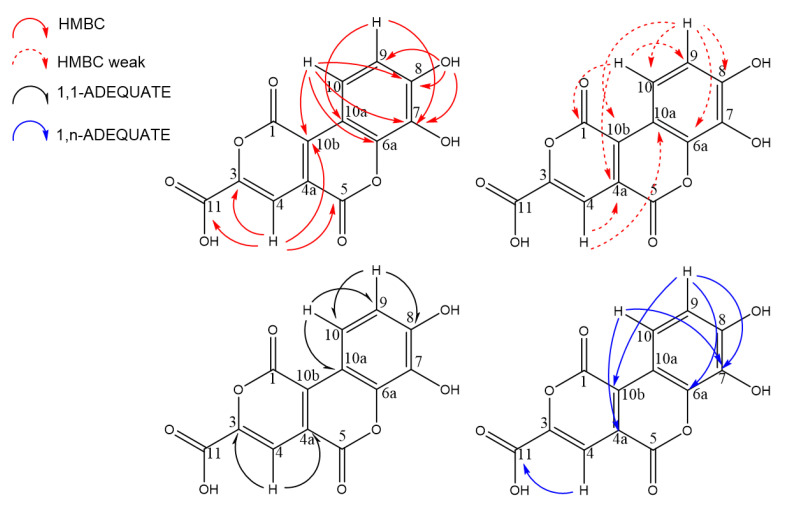
Crucial 2D HMBC (red: due to a large number of correlations, they are shown on two formulae), 1,1 ADEQUATE (black), and 1,n-ADEQUATE (blue) NMR correlations for lumnitzeralactone (**1**).

Derivatization of the molecule to incorporate additional protons (e.g., by methylation of the hydroxyl groups and formation of a methyl ester of the carboxyl group) was not performed to avoid wasting the compound without obtaining decisive information. Consequently, non-destructive methods were preferred. All attempts for crystallization, as described for molecules with related structural elements [40,42,43], did not lead to crystals suitable for X-ray analysis. Thus, further elucidation strategies relied on more unusual 2D NMR experiments, such as ^13^C-^13^C-INADEQUATE, 1,1-ADEQUATE, and 1,n-ADEQUATE, that require very high-field NMR instruments. ^13^C-^13^C-INADEQUATE provides correlations for each carbon atom with the adjacent carbon atoms through ^1^J_CC_ coupling. For molecules with a natural ^13^C abundance, the sensitivity of this 2D NMR experiment is very low due to the ^13^C-^13^C spin coupling ratio of just 0.012%. Therefore, a high sample concentration or ^13^C enrichment is recommended [44]. Because these requirements could not be met, ^13^C-^13^C-INADEQUATE experiments with a measurement time of 3 days did not provide a spectrum that showed visible correlations. Therefore, the 1,1-ADEQUATE experiment was performed, which shows pseudo ^2^J_CH_ correlations which can be used to assign the neighboring carbon atoms of proton-bearing carbons in the carbon skeleton [45]. The 1,1-ADEQUATE correlations from H-9 to C-8 and C-10 and from H-10 to C-9 and C-10a allowed for the assignment of C-8 at δ_C_ 150.8 and the aromatic carbon C-10a (δ_C_ 108.1) (Figure S2-12). Correlations from H-4 lead to the assignment of the neighboring carbons C-4a (δ_C_ 125.1) and C-3 (δ_C_ 145.8) (Figure 3).

The 1,n-ADEQUATE experiment provides information about the long-range carbon–carbon connectivity under natural abundance conditions [46], primarily via pseudo ^4^J_CH_ (^1^J_CH_ + ^3^J_CC_) correlations and occasional observations of ^3^J_CH_ (^1^J_CH_ + ^2^J_CC_) correlations [47,48]. Compared to the usual INADEQUATE experiment, sensitivity can be increased up to a factor of 64, making this approach applicable to smaller amounts of sample material [49]. In addition to the most common ^3^J_CC_ and occasional ^2^J_CC_ correlations, 2D 1,n-ADEQUATE also displays ^1^J_CC_ correlations [48,50] similar to those observed in the 1,1-ADEQUATE spectrum. The inversion of ^1^J_CC_ correlations [48,50] that leak into the 1,n-ADEQUATE spectrum would facilitate unambiguous discrimination between ^1^J_CC_ correlations (blue contours, Figure S2-11) and ^n^J_CC_ correlations (red contours, Figure S2-11). Thus, 1,n-ADEQUATE can be used to obtain both ^1^J_CC_ and ^n^J_CC_ correlations in a single experiment. However, it should be noted that 1,n-ADEQUATE is less sensitive compared to 1,1-ADEQUATE.

The combined evaluation of HMBC ^3^J_CH_ and long-range 1,n-ADEQUATE ^4^J_CH_ correlations from H-9 and H-10 to C-6a (δ_C_ 142.8) and C-7 (δ_C_ 132.7) leads to their assignment (Figure 3 and Figure S2-11). C-10b is a special case because this carbon exhibited HMBC correlations to all three protons (H-4, H-9, and H-10). H-10 and H-4 both show strong HMBC correlations to C-10b, indicating ^3^J_CH_ correlations. Although H-9 only shows a weak HMBC correlation to C-10b, the observed 1,n-ADEQUATE correlation suggested its ^4^J_CH_ coupling. The assignment of the three COOR carbons was more complicated. The correlation observed in 1,n-ADEQUATE from H-4 to the carbon at δ_C_ 160.0 (C-11) is a ^3^J_CH_ correlation, which was further supported by a strong HMBC correlation. The ^13^C spectrum of **1**, obtained in DMSO-d6, displayed two unresolved carbonyl carbons at δ_C_ 158.2 and 158.3. Thus, the correlation from H-4 to the two carbons at δ_C_ ~158 in 1,n-ADEQUATE could either be a ^3^J (H4-C5) or ^4^J (H4-C1) correlation, yet it is impossible to distinguish them. While the carbon signals at δ_C_ 158.2 and 158.3 could belong to C-1 or C-5 under the discussed conditions, both signals were better resolved in CD_3_OD (Tables S1-2 and S1-3). Here, the strong HMBC correlations of H-4 were interpreted as ^3^J_CH_, which allowed for the assignment of C5.

To localize the hydroxyl groups, a low-temperature HMBC experiment was accomplished in CD_3_OH at −20 °C (Figure S2-13) analogous to the strategy used by Vemulapalli et al., which was successfully applied to elucidate the structure of phenanthroperylene quinone pigments [51]. In this special NMR solvent and condition, the hydroxyl group of methanol is not deuterated. Consequently, the hydroxyl protons of the compound cannot be exchanged with a deuterium and remain visible as sharp signals. The hydroxyl proton at δ_H_ 10.62 (brs) shows correlations to C-7, C-8, and C-9, locating its position at C-8; another hydroxyl proton (δ_H_ 9.56, brs) did not show any correlations. This could be caused by low signal-to-noise ratios observed in the 2D NMR spectra, low sample concentration, or hardware limitations such as low observation frequencies or poorly performing probe technologies [52]. However, the location of the second hydroxyl group could be indirectly assigned to C-7 by HMBC correlations from H-9 and H-10 to C-7. An overview of all recorded HMBC spectra is shown in Table S1-3 and Figure S2. By combining all the obtained data, the structure of **1** was identified as 7,8-dihydroxy-1,5-dioxo-1,5-dihydropyrano[4,3-c]chromene-3-carboxylic acid (Figure 3). In reference to the source genus *Lumnitzera*, **1** was given the trivial name lumnitzeralactone.

### 2.3. Computer-Assisted Structure Elucidation (CASE)

To verify the structure of **1**, a CASE system was applied: the structure elucidator of Advanced Chemistry Development software (ACD-SE). All available NMR data and the molecular formula, though no predefined structural elements, were used to build the information set used as a basis for the calculation. Although it is possible to generate a “*Found Fragments*” (FF) library, especially for proton-deficient molecules, the calculations were performed in *common mode* to obtain unbiased results. After a long calculation time of more than eighteen hours, ACD-SE delivered the surprisingly low number of 44 structural proposals. Usually, more than 90% of the test sets could be calculated in less than thirty minutes, although there are cases with calculating times of more than 48 h and an output of more than 500 proposals [53]. However, even expert-challenging molecules often take just minutes to calculate when a good spectra information set (2D, 1,1-ADEQUATE) is provided, as was done here as well [53,54]. Remarkably, in the calculation results, the proposed structure for **1** was mentioned eight times, with slightly different ^13^C annotations for the COOR carbons (C-1, C-5, C-11) and for two aromatic carbons with a single oxygen bond (C-6a, C-7). The ranking of most probable structure proposals is based on d_N_(^13^C+^1^H), the average differences between predicted and experimental chemical shifts. This ranking confirmed our structure annotation for lumnitzeralactone (**1**). The proposal with the correct carbon annotation was listed as the first ranked hit with a d_N_ of 3.888. Furthermore, the correct structure appeared at positions 2 to 4, as well as at positions 7, 8, 11, and 12 (for the whole ranking, see Figure S8).

### 2.4. Density Functional Theory (DFT) Calculations

**Figure 4:**
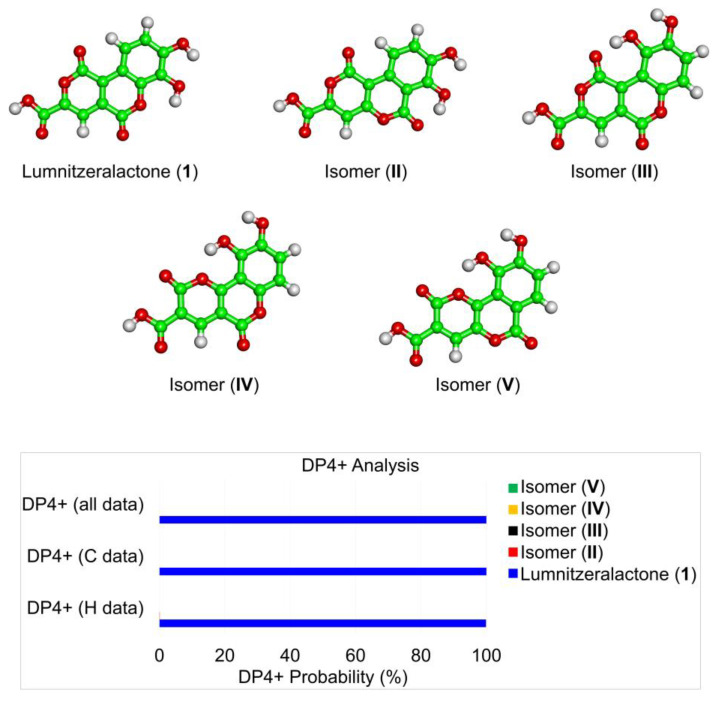
The proposed molecular structures of lumnitzeralactone (**1**), its alternative isomeric structures, and their computed DP4+ probabilities using ^1^H and ^13^C NMR data. The DP4+ probabilities were obtained by correlating the experimental ^1^H and ^13^C NMR data with the calculated (PCM/mPW1PW91/6-311+G(d,p)//B3LYP/6-31+G(d,p)) nuclear shielding tensors. Compound **1** is unambiguously confirmed as the correct structure of the isolated compound through DP4+ probability (blue bars) for ^1^H (99.89%), ^13^C (100%), and all data (100%). Isomeric structures (II-V) show a probability of 0%.

In addition, the structure of **1** was verified by DFT calculations. Five potential structural isomers of **1** (lumnitzeralactone and isomers II-V) were considered for this computational quantum mechanical modelling (Figure 4 and Table S2-1). For each structure, only one dominant conformer was obtained (Table S2-2). For these, the experimental and calculated chemical shifts were compared. The anticipated structure of lumnitzeralactone (**1**) is assigned a very high probability by ^1^H-DP4+ (99.89%), ^13^C-DP4+ (100%), and (^1^H + ^13^C)-DP4+ (100%), while the alternative structures (isomers II-V) are assigned a probability of almost 0%. Thus, the proposed structure for lumnitzeralactone (**1**) could be unambiguously identified as the correct structure.

### 2.5. Synthesis

**Scheme 1:**
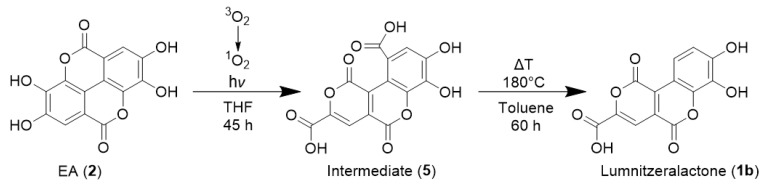
Two-step chemical synthesis of lumnitzeralactone (**1b**) from EA (**2**) via formation of a stable intermediate (**5**).

A further proof of the structure of **1** was obtained by chemical synthesis, something which was required for final structural proof for many natural products [55,56]. The synthesis of **1** was achieved in a two-step reaction starting from **2**, as shown in Scheme 1. The first step is the photo-oxidation of **2**, which produces intermediate **5** that has a similar structure to the natural product **1**. Following the protocol of Tokutomi et al. [43] who first described this reaction, we could obtain a good yield of the desired intermediate **5** under adapted conditions. To improve conversion, the reaction was conducted under oxygen atmosphere in an ice bath to avoid overheating from the lamp. All recorded NMR (Figure S3, Table 1) and HRMS data (Table 2, Figure S7) of **5** are in accordance with data reported in the literature [43]. For the second step, Cu-catalyzed and Ag-catalyzed protodecarboxylation was first attempted for a selective decarboxylation of aromatic carbonic acids [57,58], though no product could be observed. However, thermal decarboxylation of **5** in toluene at 180 °C yielded the desired singly decarboxylated product **1b,** as well as side products. NMR data (Table 1, Figure S4) and HRMS data (Table 2, Figure S6) of the synthesized lumnitzeralactone (**1b)** are in full accordance with data from the natural lumnitzeralactone (**1**).

### 2.6. Biosynthetic Considerations

Compound **1** was found in only two of the 31 investigated *Lumnitzera* samples [8,34]. Therefore, it is likely that biosynthesis of the natural product is not (exclusively) dependent on the plant host, which shows low levels of genetic variation at a population level [59]. Instead, biosynthesis is the result of interactions with associated microorganisms that can highly depend on local environmental conditions. We suggest fungal participation in the transformation of **2** to **1** through an enzymatic process. In a fungal fermentation experiment by Aguilar-Zárate et al., an unknown EA degradation product with the same *m/z* as **1** was detected [60]. One possible biosynthetic pathway could begin with radical-triggered (oxidative) decarboxylation, performed by an oxidative enzyme originating from associated fungi. Subsequent steps include further oxidation and cyclization (Scheme S2-1). Several oxidizing enzymes are known from fungi, many of which exhibit extracellular activity and act on polyphenols [61,62,63,64,65,66]. Decarboxylating enzymes of fungal origin are involved in the degradation of lignin [67,68,69] and gallo- and ellagitannins [70].

**Scheme 2:**
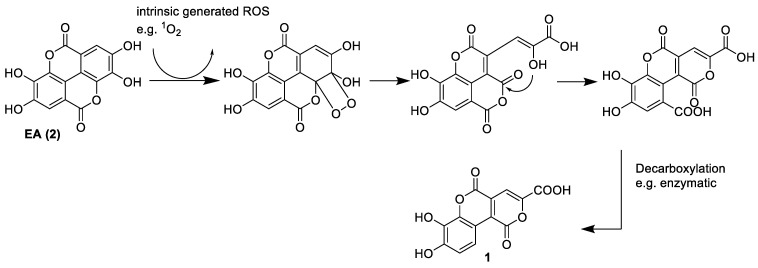
A suggested pathway for the biosynthesis of lumnitzeralactone (**1**), including ROS (analogous to Tokutomi et al. [43], cf. Scheme 1).

Therefore, we suggest an alternative pathway analogous to chemical synthesis [43] via the intermediate **5**. The endoperoxide intermediate might be formed by cycloaddition of ROS [43] or enzymatically by an oxygen incorporating enzyme such as dioxygenase. This is followed by enzymatic decarboxylation (Scheme 2). However, **1** is found in the root bark, and we have no evidence at this point as to whether it is formed only superficially or by root penetrating or endophytic fungi and how the transport of EA (**2**), EA derivatives, or lumnitzeralactone (**1**) occurs between species.

### 2.7. Biological Activity

Since **1** was detected exclusively in the two anti-bacterial crude extracts [8,34], the contribution of **1** to this activity was hypothesized. Thus, the anti-bacterial activity of **1** was checked. However, in contrast to expectations, **1** did not inhibit bacterial growth (Table 3). Interestingly, fractions resulting from the purification process and containing mainly **1** showed anti-bacterial effects (95% inhibition at a concentration of 500 µg/mL, Table 3). Analysis of the metabolites in this active fraction revealed, besides **1,** the presence of **3** (Figure S10). In accordance with reports in the literature [71], **3** exhibited a clear anti-bacterial effect in the assay with an inhibition rate of nearly 100% at a concentration of 100 µM (Table 3). However, **3** does not seem to be responsible for the observed anti-bacterial activity profile of the mangrove extracts, as this compound was detected in the majority of the 31 *Lumnitzera* crude extracts addressed in our previous comprehensive profiling study and its occurrence did not correlate with the effects [8]. At this moment, we can only speculate that a synergistic effect might contribute to the observed anti-bacterial effects, or that the other extracts had matrix effects countered by compound **1**.

As mentioned above, mangroves often live in symbiosis with associated microorganisms, including fungi. An intensive investigation of endophytic fungi from mangroves, including *Lumnitzera*, revealed significant anti-microbial potential for 71 representative endophytic fungal species tested. Their extracts were applied against a set of two Gram-positive bacteria (*B. subtilis* and *S. aureus*) and two Gram-negative bacteria (*Pseudomonas aeruginosa* and *Escherichia coli*) [32]. Consistent with this study, our results imply that EA-metabolizing fungi might contribute to the anti-bacterial effects of selected *Lumnitzera* samples.

## 3. Material and Methods

### 3.1. General Experimental Procedures and Reagents

Thin layer chromatography (TLC) analyses were performed on silica gel 60 reversed-phase 18 F_254_ (Merck, Darmstadt, Germany) using the solvent system H_2_O:MeOH 3:2 or silica gel 60 reversed-phase 2 UV_254_ (Macherey-Nagel, Düren, Germany) using the solvent system H_2_O:MeOH 3:2. To visualize the compound spots, long-wavelength UV light (366 nm), short-wavelength UV light (254 nm), and spraying with vanillin-H_2_SO_4_ reagent were used, followed by heating or spraying with a natural product spray reagent.

Low-resolution ESI-MS spectra were obtained using a Sciex API-3200 instrument (Applied Biosystems, Concord, ON, Canada) combined with an HTC-XT autosampler (CTC Analytics, Zwingen, Switzerland).

The UV spectra were recorded on a Jasco V-770 UV-Vis/NIR spectrophotometer (Jasco, Pfungstadt, Germany) using a 10 mm quartz glass cuvette.

Analytical and semi-preparative RP-HPLC were performed on a Shimadzu prominence system consisting of an SPD-M20A diode array detector, a FRC-10A fraction collector, a CBM-20A communications bus module, a DGU-20A5R degassing unit, an LC-20AT liquid chromatograph, and an SIL-20A HT auto sampler. Chromatographic separation was performed using an analytical YMC Pack Pro C18 column (ID 4.6 mm, length 150 mm, particle size 5 µm) and a semi-preparative YMC Pack Pro C18 column (ID 10.0 mm, length 150 mm, particle size 5 µm) using ultrapure water (TKA ultrapure water system) and methanol (Merck, LiChrosolv HPLC Gradient Grade) as eluents.

Ellagic acid was purchased from TCI Chemicals (Tokyo, Japan) and was used without further purification. All solvents were purchased from Merck Chemicals GmbH (Darmstadt, Germany) and were distilled prior to use. Deuterated solvents for NMR spectroscopy were purchased from Deutero GmbH (Kastellaun, Germany). TMEA (**3**) was obtained by earlier isolation [8].

### 3.2. Plant Material

The root material of *Lumnitzera racemosa* Willd. was collected from the Indonesian archipelago as described in Manurung et al. [8] in Table 1, No. 19. The material corresponding to sample LR7 comes from Ternate Island, Maluku (DD coordinates 0.84 /127.31). The voucher specimen (BO1959402) is deposited at Herbarium Bogoriense (BO, Bogor, Indonesia), National Research and Innovation Agency (BRIN). The samples were cleaned, air-shadow-dried, and then kept in resealable zipper storage bags until use for further treatment.

### 3.3. Extraction and Isolation

An aliquot (1.35 g) of the crude extract used in previous work [8] was diluted in 200 mL water and extracted five times with 100 mL of ethyl acetate. Each ethyl acetate fraction was centrifuged. The combined supernatant of fractions 2–5 was dried (49.2 mg) and submitted to an RP18 column eluted with a mixture of water and methanol (30:20, *v/v*) followed by final purification by preparative HPLC (water (A)/methanol (B) gradient: 0–17.5 min, 5–31.5% B; 17.5–19.5 min, 31.5–100% B, isocratic for 8 min and a flow rate of 0.8 mL/min) to yield lumnitzeralactone (**1**) (2.5 mg, Rf = 0.71 in MeOH/H_2_O (2:3, *v/v*) on RP18).

For repeated isolation, 15.06 g of dried roots from *L. racemosa* was ground to fine powder in a ball mill, followed by an exhaustive extraction with methanol to provide 1.33 g of dried crude extract. The extract was partitioned by liquid–liquid extraction between water and ethyl acetate, first pure, then by adding some drops of 2M HCl to the water phase, resulting in three fractions: water (697.2 mg), ethyl acetate (pure) (312.8 mg), and acidic ethyl acetate (75.5 mg).

The last fraction was separated using a Sephadex LH20 column (h: 76 cm, d: 2.5 cm) eluted with pure methanol. Based on TLC profiles, six main fractions were combined. Fraction 3 (Rf = 0.72) was further purified on an RP2 cartridge (h: 5.5 cm, d: 1.6 cm) and eluted with a mixture of 40% methanolic water followed by 10% methanolic chloroform solution. Final purification of the water–methanol fraction was performed by preparative HPLC. Compound **1** (14.6 mg, Rf = 0.72 in MeOH/H_2_O (2:3, *v/v*) on RP18) was purified using a water (A)/methanol (B) gradient system (0–17.5 min, 5–31.3% B; 17.5–19.5 min, 100% B (isocratic for 8 min)) and a flow rate of 7.089 mL/min at 25 °C with absorbance detection at 210 to 800 nm (R_t_ = 9.038 min, λ_max_: 411 nm, 288 nm, 210 nm).

The pure ethyl acetate fraction resulting from the liquid–liquid extraction was separated in the same way using an LH20 column and an RP2 cartridge to obtain a fraction containing **1** and TMEA (**3**) (54.0 mg). This fraction was used in the anti-bacterial assay.

Lumnitzeralactone (7,8-dihydroxy-1,5-dioxo-1,5-dihydropyrano[4,3-c]chromene-3-carboxylic acid, **1**): yellow amorphous solid; UV (THF) λ_max_ (log ε) 232 (4.10), 290 (3.95), 407 (3.96) nm; ^1^H and ^13^C NMR, see Table 1; HR ESI-MS and MS^2^ fragmentation, see Table 2.

### 3.4. Synthesis

#### 3.4.1. Photoreaction

A solution of ellagic acid dihydrate (0.82 g, 2.4 mmol) in dry THF (800 mL) was irradiated using a mercury xenon lamp for 45 h inside a photoreactor. The reaction vessel was placed in an ice bath for additional cooling and a balloon filled with oxygen was attached to it. Reaction progress was monitored through measurement of UV spectra to detect the decreases in absorption intensity at 367 nm and increases in intensity at 400 nm, as performed by Tokutomi et al. [43]. After completion of the reaction, the solvent was distilled off and the residue was freeze-dried. The crude product was suspended in DCM and then stored overnight in the refrigerator. Subsequently, the supernatant was removed, and the precipitation step was repeated, yielding 0.79 g of an orange amorphous solid (**5**) which was used without further chromatographic purification.

Synthesized intermediate of lumnitzeralactone (7,8-dihydroxy-1,5-dioxo-1,5-dihydropyrano[4,3-c]chromene-3,10-dicarboxylic acid, **5**): orange amorphous solid; UV (THF) λ_max_ (log ε) 232 (4.13), 290 (3.77), 407 (3.63) nm; ^1^H and ^13^C NMR, see Table 1; HR ESI-MS and MS^2^ fragmentation, see Table 2.

#### 3.4.2. Decarboxylation

A solution of intermediate **5** (100 mg) in dry toluene (2 mL) was heated to 180 °C in a capped microwave vial for 60 h. After distilling off the solvent, the reaction product was dissolved in methanol and centrifuged to separate insoluble residues. The supernatant was purified using an RP18 column (h: 36 cm, d: 3.5 cm) and eluted with a water–methanol mixture (3:2, *v/v*) to obtain synthetic lumnitzeralactone (**1b**) (6.7 mg, 0.023 mmol, 7.5% yield over two steps).

Synthetic lumnitzeralactone (**1b**): yellow amorphous solid; UV (THF) λ_max_ (log ε) 232 (4,01), 290 (3.94), 411 (3.98) nm; ^1^H and ^13^C NMR, see Table 1; HR ESI-MS and MS^2^ fragmentation, see Table 2.

### 3.5. NMR

^1^H and ^13^C NMR spectra were recorded on an Agilent DD2 400 NMR spectrometer at 399.917 and 100.570 MHz, respectively. Chemical shifts are reported relative to TMS (^1^H NMR) or solvent peaks (^13^C, DMSO-*d6* 39.5 ppm, MeOH-*d4* 49.0 ppm). For samples with low concentrations, ^1^H and ^13^C NMR spectra were recorded on a Bruker Avance Neo 500 NMR spectrometer at 500.234 and 125.797 MHz, respectively, using a 5 mm prodigy probe with TopSpin 4.0.7 spectrometer software. The 2D NMR spectra were recorded on an Agilent VNMRS 600 MHz NMR spectrometer using standard CHEMPACK 8.1 pulse sequences (^1^H-^13^C gHSQCAD, ^1^H-^1^H gCOSY, ^1^H-^1^H gTOCSY, and ^1^H-^13^C gHMBCAD) implemented in Varian VNMRJ 4.2 spectrometer software.

The low-temperature ^13^C-^1^H long-range correlation HMBC spectrum for structure elucidation of **1** was recorded at 253 K on a Bruker Avance NEO 700 MHz (^1^H resonance frequency) instrument equipped with a 5 mm TCI cryoprobe prodigy. Long-range carbon–proton coupling of 8 Hz was used. The time domain matrix of 4k × 256 with 13 ppm (F2) and 80 ppm (F1) spectral width was used. Carrier frequency was set to 5.5 and 135 ppm in the F2 and F1 dimensions, respectively. The number of scans was set to 256 per t1 increment and 2 s of repetition delay was used. The 1,1-ADEQUATE [72] (Bruker pulse sequence: adeq11etgpsp) spectrum was recorded on a Bruker Avance III HD 900 MHz (^1^H resonance frequency) instrument equipped with a 5 mm TCI cryoprobe. One-bond carbon–proton and carbon–carbon couplings of 145 and 50 Hz, respectively, were used. The inversion (Crp60, 0.5, 20.1) and refocusing (Crp60, 0.5, 20.1) 180° selective pulses on ^13^C were set to 500 and 2000 µs, respectively. The time domain matrix of 3k × 208 with 13 ppm (F2) and 147 ppm (F1) spectral width was used. Carrier frequency was set to 6 and 100 ppm in the F2 and F1 dimensions, respectively. The number of scans was set to 256 per t1 increment and 2 s of repetition delay was used. The 1,n-ADEQUATE [48,50,72,73] (Bruker pulse sequence: adeq1netgprdsp) spectrum was recorded on a Bruker Avance NEO 800 MHz (^1^H resonance frequency) instrument equipped with a 3 mm TCI cryoprobe. One-bond carbon–proton and carbon–carbon couplings of 145 and 57 Hz (64 Hz), respectively, were used. The desired long-range carbon–proton coupling was set to 9.5 Hz (8 Hz). NMR spectra were processed and analyzed using Topspin 4.1.3 (Bruker, Germany).

### 3.6. UHPLC-ESI-QqTOF-MS and MS/MS

For mass spectra of pure compounds, the samples (2 µL) were loaded on an EC 150/2 Nucleoshell RP 18 column (C18-phase, ID 2 mm, length 150 mm, particle size 2.7 µm, Macherey Nagel, Düren, Germany) under isocratic conditions (3% eluent B, 1 min) and separated using a linear gradient from 3% to 95% eluent B in 5 min. Separation was performed on an ACQUITY UPLC I-Class UHPLC System (Waters GmbH, Eschborn, Germany) with a flow rate of 0.4 mL/min and 55 °C column temperature. Eluents A and B were water and acetonitrile, with 0,1% (*v/v*) formic acid. The column effluent was introduced online into a TripleTOF 6600 quadrupole time-of-flight (QqTOF) mass spectrometer equipped with a DuoSpray ESI/APCI ion source operating in negative ion SWATH (Sequential Windowed Acquisition of All Theoretical Fragment Ion Mass Spectra) mode and controlled by Analyst TF 1.7.1 software (AB Sciex GmbH, Darmstadt, Germany). The TOF scans (MS experiments) were acquired in the *m*/*z* range of 50 to 1000 (accumulation time 50 ms) with an ion spray voltage of −4.5 kV and 450 °C source temperature. Declustering (DP) and collision (CE) potentials were −35 and −10 V, respectively. The product ion spectra (tandem mass spectra, MS/MS) were acquired in the high sensitivity mode (accumulation time 20 ms) in the *m*/*z* range of 50–350 using unit Q1 resolution with mass resolution above 30,000. Collision potential (CE) was set from −80 to −20 V, whereas collision energy spread (CES) was 15 V. The data were evaluated by Peak View 1.2.0.3 software (AB Sciex GmbH, Darmstadt, Germany).

The crude extract was investigated by applying the MS conditions described in Manurung et al. [8].

### 3.7. DFT-Calculations

The starting structures representing potential isomers of **1** were built in Maestro 11.4 (Schrödinger Release 2017-4: Maestro; Schrödinger, LLC: New York, NY, USA, 2019.). The conformational search was performed with Macromodel 11.8 (Schrödinger Release 2017-4: Macromodel; Schrödinger, LLC: New York, NY, USA, 2019) using the MMFF forcefield [74] under vacuum and an energy threshold of 5 kcal/mol. Only one dominant conformer was obtained for each structure. All conformers were geometry-optimized at the B3LYP [8,9,10,11]/6-31+G(d,p) [75] level of theory with Gaussian09 (Gaussian 09, Revision C.01; Gaussian Inc.: Wallingford, CT, USA, 2010). The energy minimized conformers were used as an input geometry for further DFT calculations. The nuclear shielding constants were calculated at mPW1PW91 [76,77] /6-311+G(d,p) and mPW1PW91/6-311+G(2d,p) levels of theory using GIAO [78] and IEFPCM [79] solvent (methanol) models. The obtained shielding constants were converted into chemical shifts using the scaling factors available on the CHESHIRE (chemical shift repository) [80,81,82,83] webpage. DP4+ [84,85] probability was obtained using the experimental and calculated ^1^H and ^13^C chemical shifts.

### 3.8. ACD-SE Calculations

The ACD/Structure Elucidator (ACD/SE) from ACD/Labs in ACD/Labs version 2018.2.5 (File Version S80S41, Build 108235, 8 April 2019) was used to perform verification of promising structure proposals based on experimental NMR and HRMS data.

### 3.9. Anti-Bacterial Assay

The compounds were evaluated against the Gram-positive *Bacillus subtilis* 168 (DSM 10), as described by Ware et al. [86]. The tests were performed in 96-well plates based on absorption read-out. Chloramphenicol (100 µM) was used as a positive control to induce complete inhibition of bacterial growth. The results (mean ± standard deviation value, *n* = 6) are given in relation to the negative control (bacterial growth in the presence of 1% *v/v* DMSO) as relative values (percent inhibition). Negative values indicate an increase in bacterial growth.

## 4. Conclusions

In this study, the previously unreported lumnitzeralactone (**1**) was isolated and characterized from the true mangrove species *Lumnitzera racemosa*. Elaborate structure elucidation includes ^1^H and ^13^C NMR, 2D NMR (COSY, TOCSY, HSQC, HMBC, 1,n-ADEQUATE, 1,1-ADEQUATE) spectra recorded in different solvents and in special cases under low-temperature conditions, HR-MS, computer-assisted structure elucidation (CASE), DFT calculations, and chemical synthesis. In contrast to expectations, lumnitzeralactone (**1**) isolated from the anti-bacterial crude extract did not exhibit significant anti-bacterial activity against *B. subtilis*.

Putative biosynthetic pathways of **1** are suggested, as well as a high probability of the participation of an associated microorganism or its excreted enzymes. Microorganism-based modification or elicitation may also explain the observed differential antibiotic potential of the same species when collected at different sites. Although **1** itself did not show significant anti-bacterial activity, it is present exclusively in anti-bacterial crude extracts. However, the activity of crude extracts can also result from yet unidentified highly bioactive minor components. Considering this, Indonesian mangroves may represent a promising source of potent bioactive compounds that are waiting to be explored further.

## Figures and Tables

**Table 1:** ^13^C (100 MHz) and ^1^H NMR (400 MHz) data of **1** and synthesized **1b** and **5**.

	Lumnitzeralactone (1)		Synthetic Lumnitzeralactone (1b)		Synthetic Intermediate (5)	
Position	δ_C_, Type DMSO-*d6*	δ_H_, m (*J* in Hz) DMSO-*d6*	δ_C_, Type DMSO-*d6*	δ_H_, m (*J* in Hz) DMSO-*d6*	δ_C_, Type ^a^ THF-*d8*	δ_H_, m (*J* in Hz) ^a^ THF-*d8*
1	158.2, C		158.3, C		158.8, C	
3	145.8, C		146.2, C		147.6, C	
4	107.3, CH	7.49, s	107.2, CH	7.48, s	107.4, CH	7.54, s
4a	125.1, C		125.1, C		124.9, C	
5	158.3, C		158.3, C		158.4,	
6a	142.8, C		142.8, C		144.2, C	
7	132.7, C		132.7, C		135.2, C	
8	150.8, C		150.7, C		149.5, C	
9	113.2, CH	6.95, d (9.0)	113.2, CH	6.94, d (9.0)	115.9, CH	7.21, s
10	117.9, CH	8.33, d (9.0)	117.8, CH	8.33, d (9.0)	127.4, C	
10a	108.1, C		108.1, C		107.2, C	
10b	127.9, C		127.8, C		130.8, C	
11	160.0, C		160.1, C		160.4, C	
12					167.8, C	
7-OH		9.56, brs		9.56, brs		9.48, brs
8-OH		10.62, brs		10.59, brs		9.34, brs

^a^ NMR data in accordance with the study conducted by Tokutomi et al. [40].

**Table 2:** Characterization of the natural lumnitzeralactone (**1**) and synthesized compounds (**1b** and **5**) through reversed-phase ultra-high-performance liquid chromatography–quadrupole time-of-flight tandem mass spectrometry (RP-UHPLC-QqTOF MS/MS).

No	*t*_R_ (min)	*m/z* [M − H]^−^ Observed	*m/z* [M − H]^−^ Calculated	Δppm	Elemental Composition	RDB	Fragmentation Patterns
**1**	2.5	289.0002	288.9990	4.2	C_13_H_6_O_8_	11.5	65.0033 (C_4_HO, 8), 117.0346 (C_8_H_5_O, 25), 133.0293 (C_8_H_5_O_2_, 14), 145.0297 (C_9_H_5_O_2_, 100), 151.0035 (C_7_H_3_O_4_, 9), 161.0244 (C_9_H_5_O_3_, 49), 173.0247 (C_10_H_5_O_3_, 28), 189.0196 (C_10_H_5_O_4_, 42), 200.0115 (C_11_H_4_O_4_, 11), 217.0145 (C_11_H_5_O_5_, 26) 228.0064 (C_12_H_4_O_5_, 14) 245.0098 (C_12_H_5_O_6_, 21)
**1b**	2.5	288.9990	288.9990	0	C_13_H_6_O_8_	11.5	65.0026 (C_4_HO, 7), 117.0326 (C_8_H_5_O, 30), 133.0288 (C_8_H_5_O_2_, 13), 145.0292 (C_9_H_5_O_2_, 100), 151.0032 (C_7_H_3_O_4_, 8), 161.0240 (C_9_H_5_O_3_, 50), 173.0239 (C_10_H_5_O_3_, 26), 189.0191 (C_10_H_5_O_4_, 40), 200.0109 (C_11_H_4_O_4_, 10), 217.0138 (C_11_H_5_O_5_, 29), 228.0025 (C_12_H_4_O_5_, 15), 245.0089 (C_12_H_5_O_6_, 24)
**5**	1.9	332.9887	332.8889	–0.4	C_14_H_6_O_10_	12.5	77.0386 (C_6_H_5_, 5), 105.0333 (C_7_H_5_O, 19), 117.0332 (C_8_H_5_O, 9), 133.0284 (C_8_H_5_O_2_, 38), 145.0282 (C_9_H_5_O_2_, 10), 161.0233 (C_9_H_5_O_3_, 100), 189.0186 (C_10_H_5_O_4_, 57), 202.9975 (C_10_H_3_O_5_, 3), 217.0134 (C_11_H_5_O_5_, 12), 233.0086 (C_11_H_5_O_6_, 13), 261.0039 (C_12_H_5_O_7_, 22)

**Table 3:** Anti-bacterial activity against Gram-positive *Bacillus subtilis* of the crude extract, a fraction (both 500 µg/mL), and pure compounds (100 µM) derived from *L. racemosa* and its synthetic analog.

Sample	Growth Inhibition [%]
**1**	11.9 ± 26.2
**1b**	21.7 ± 11.4
**5**	28.9 ± 10.6
**3**	99.9 ± 3.0
Fraction containing **1** and **3**	94.6 ± 7.1
Crude extract	90.1 ± 20.6
Pos. control (Chloramphenicol)	98.9 ± 0.1

## Data Availability

Plant material: Plant material is deposited at LIPI, Bogor, Indonesia. Chemical data: All primary data and reference compounds are stored in IPB primary data storage for 10+ years and in the compound depository to the extent available or stable. Pending availability, detailed data can be shared upon request.

## Supplementary Materials

The following supporting information can be downloaded at: https://www.mdpi.com/article/10.3390/md21040242/s1, Figure S1: TLC after Bornträger reaction, Figure S2: The 1D and 2D NMR spectra of compound **1**, Figure S3: The 1D and 2D NMR spectra of compound **5**, Figure S4: The 1D and 2D NMR spectra of compound **1b**, Figure S5: MS data of compound **1**, Figure S6: MS data of compound **1b**, Figure S7: MS data of compound **5**, Figure S8: Structure Elucidation Report—ACD-SE Calculation, Figure S9: UV spectra of compound **1**, **1b**, and **5**, Figure S10: ^1^H NMR spectrum and HPLC chromatogram of the anti-bacterial fraction containing **1** and **3**, Scheme S1: Mechanism of the Bornträger reaction, Scheme S2: Suggested pathway for the biosynthesis of compound **1**, Table S1: ^1^H, ^13^C, and HMBC NMR data of compound **1** with different solvents and field strengths, Table S2: Additional data relating to DFT calculations.
