# Supplementary material for: Challenging Structure Elucidation of Lumnitzeralactone, an Ellagic Acid Derivative from the Mangrove Lumnitzera racemosa
Source: Mar Drugs. 2023 Apr 14;21(4):242. doi: 10.3390/md21040242 (PMC10144801; doi:10.3390/md21040242)
Supplement: Supplementary file 1 [file marinedrugs-21-00242-s001.zip › marinedrugs-2317992-supplementary.pdf]

Supporting Information

Challenging structure elucidation of lumnitzeralactone, an ellagic acid derivative from the mangrove *Lumnitzera racemosa*

Jonas Kappen[1], Jeprianto Manurung[1,2,3], Tristan Fuchs[1], S. Phani B. Vemulapalli[4,5], Lea M. Schmitz[1], Andrej Frolov[1], Andria Agusta[6], Alexandra N. Muellner-Riehl[2,3], Christian Griesinger[4]*; Katrin Franke[1,3,7]* and Ludger A. Wessjohann[1,3]*

[1] Department of Bioorganic Chemistry, Leibniz Institute of Plant Biochemistry (IPB), 06120 Halle (Saale), Germany; jkappen@ipb-halle.de (J.K.); tfuchs@ipb-halle.de (T.F.); lschmitz@ipb-halle.de (L.M.S.); andrej.frolov@ipb-halle.de (A.F.); wessjohann@ipb-halle.de (L.A.W.); kfranke@ipb-halle.de (K.F.)
[2] Department of Molecular Evolution and Plant Systematics & Herbarium (LZ), Institute of Biology, Leipzig University, 04103 Leipzig, Germany; jeprianto_m@apps.ipb.ac.id (J.M.); muellner-riehl@uni-leipzig.de (A.N.M.R.)
[3] German Centre for Integrative Biodiversity Research (iDiv) Halle-Jena-Leipzig, 04103 Leipzig, Germany
[4] Department of NMR-Based Structural Biology, Max Planck Institute for Multidisciplinary Sciences, Am Fassberg 11, 37077 Göttingen, Germany; save@mpinat.mpg.de (S.P.B.V.); cigr@mpinat.mpg.de (C.G.)
[5] Research Group for Marine Geochemistry, Institute for Chemistry and Biology of the Marine Environment (ICBM), Carl von Ossietzky Universität Oldenburg, Carl-von-Ossietzky-Str. 9-11, 26129 Oldenburg, Germany
[6] Research Center for Pharmaceutical Ingredients and Traditional Medicine, National Research and Innovation Agency (BRIN), Jl. M.H. Thamrin No. 8, Jakarta 10340, Indonesia; andr005@brin.go.id (A.A.)
[7] Institute of Biology/Geobotany and Botanical Garden, Martin Luther University Halle-Wittenberg, Halle, Germany

* Correspondence: cigr@mpinat.mpg.de, Tel: +49 551 201-2201 (C.G.); kfranke@ipb-halle.de, Tel: +49-345-5582-1380 (K.F.); wessjohann@ipb-halle.de, Tel: +49-345-5582-1301 (L.A.W.)

Content **page**

Figure S1: TLC after the Bornträger-reaction 3

Scheme S1: Putative mechanism of the Bornträger reaction 3

Figure S2-1–S2-13: 1D and 2D NMR spectra of compound **1** 4

Table S1-1–S1-3: ^{1}H, ^{13}C and HMBC data of compound **1** in different solvents and field strengths 11

Figure S3-1–S3-4: 1D and 2D NMR spectra of compound **5** 12

Figure S4-1–S4-5: 1D and 2D NMR spectra of compound **1b** 14

Figure S5-1–S5-2: MS data of compound **1** 16

Figure S6-1–S6-2: MS data of compound **1b** 17

Figure S7-1–S7-2: MS data of compound **5** 18

Table S2-1–S2-2: Additional data DFT-Calculations 20

Figure S8-1–S8-3: Structure elucidation report - ACD-SE-Calculation 24

Scheme S2-1: Suggested pathway for the biosynthesis of compound **1** 27

Figure S9-1–S9-2: UV spectra of compound **1**, **1b** and **5** 28

Figure S10-1–S10-2: ^{1}H NMR spectrum and HPLC chromatogram of the fraction containing **1** and **3** 29

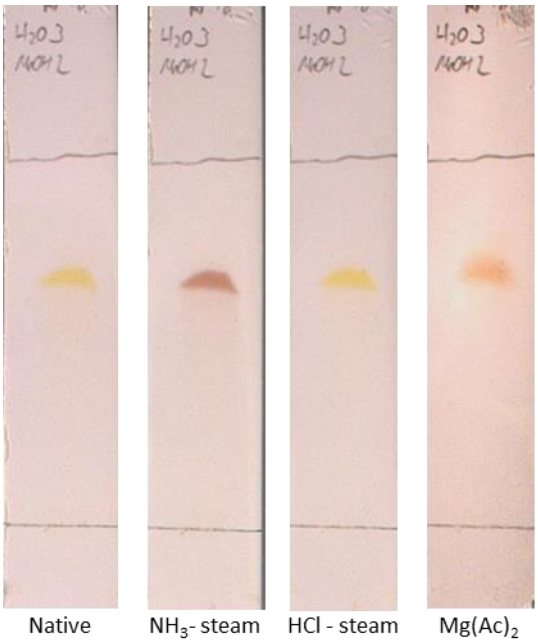

Figure S1. TLC of lumnitzeralactone (**1**) after the Bornträger reaction; stationary phase: RP18, solvent system: H_2O:MeOH, 3:2 v/v.

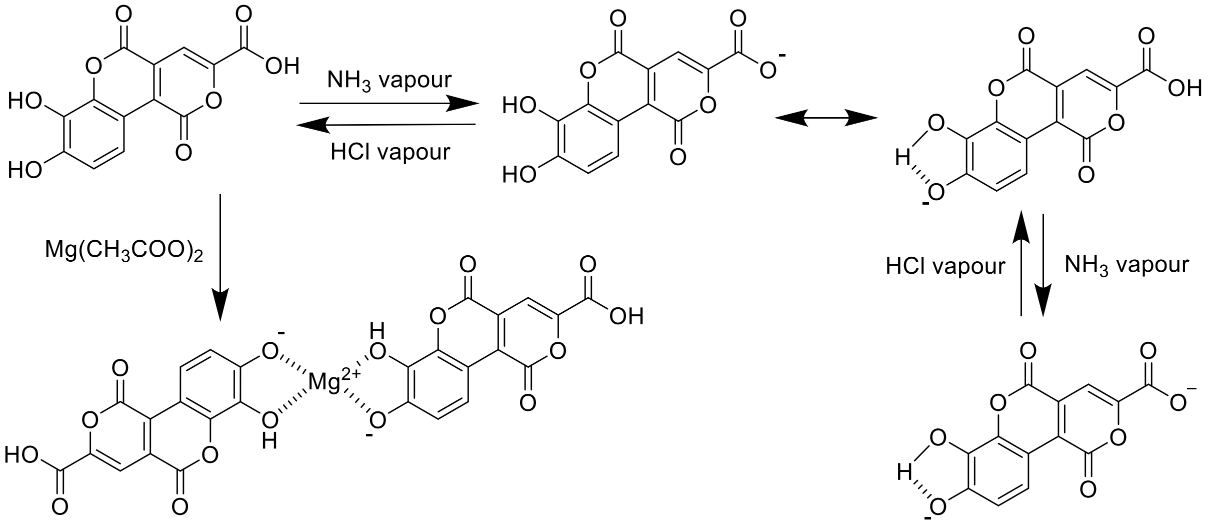

Scheme S1. Putative mechanism of the Bornträger reaction for lumnitzeralactone (**1**)

Lumnitzeralactone (**1**)

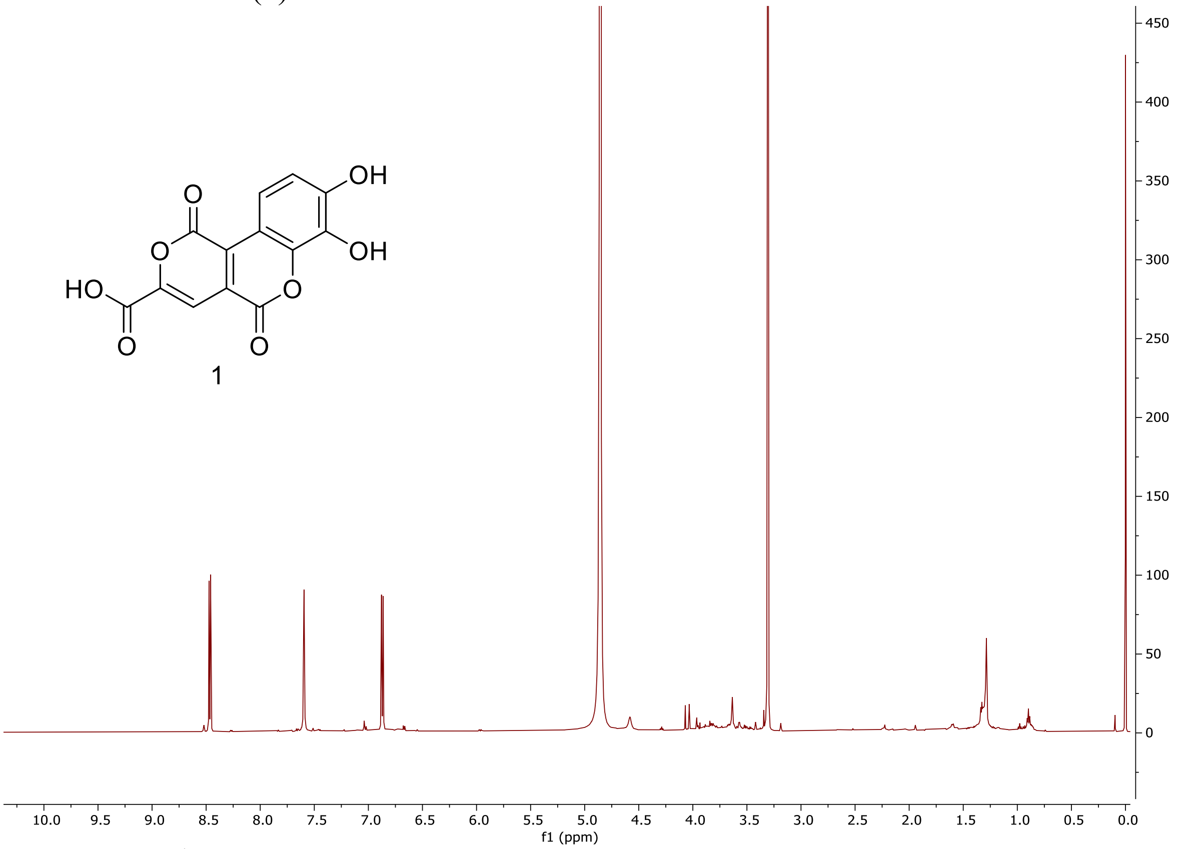

Figure S2-1. ^{1}H NMR spectrum of compound **1** in MeOH-*d4*, 600 MHz

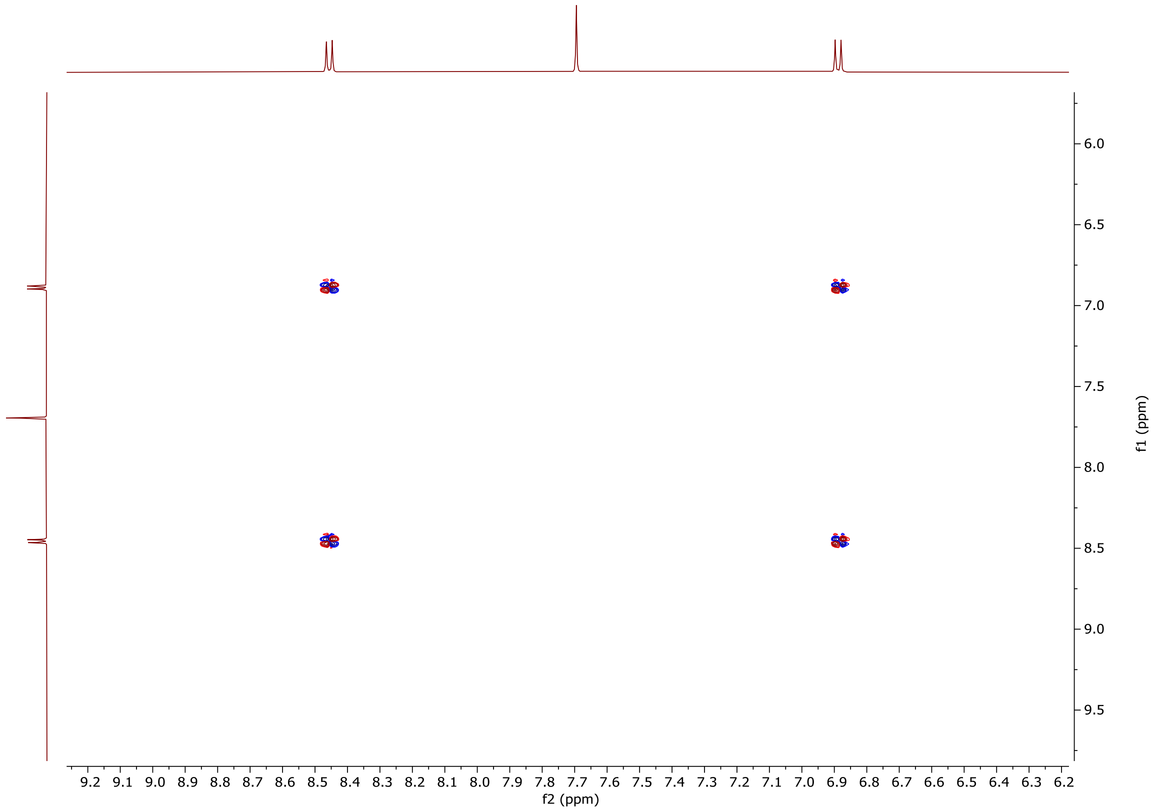

Figure S2-2. COSY spectrum of compound **1** in MeOH-*d4*, 500 MHz

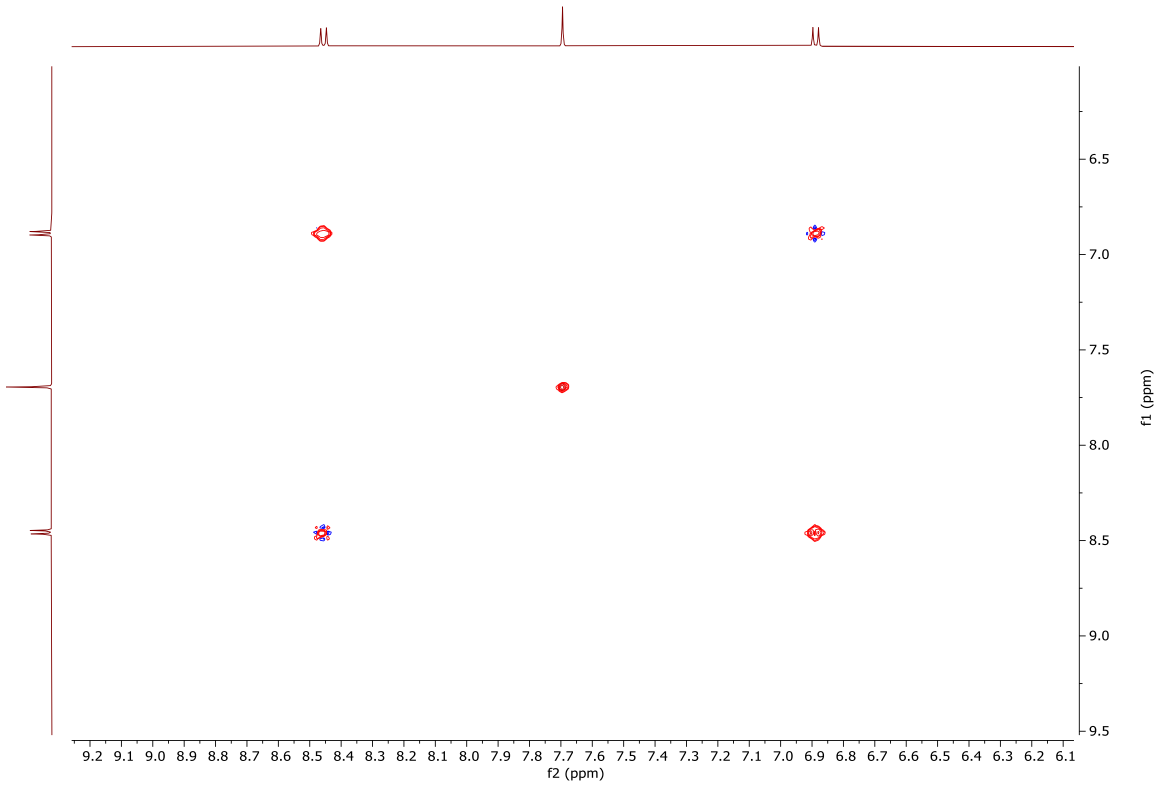

Figure S2-3. TOCSY spectrum of compound **1** in MeOH-*d4*, 500 MHz

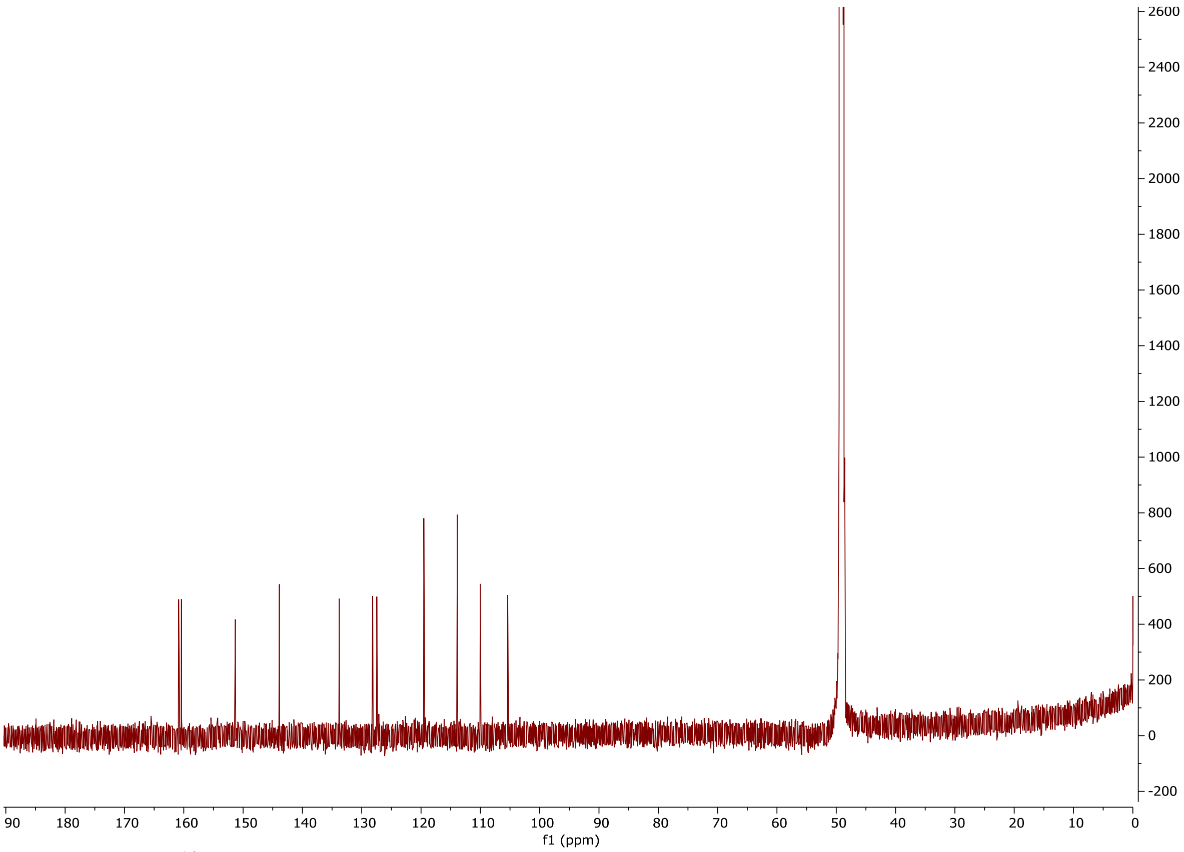

Figure S2-4. ^{13}C-NMR spectrum of compound **1** in MeOH-*d4*, 150 MHz

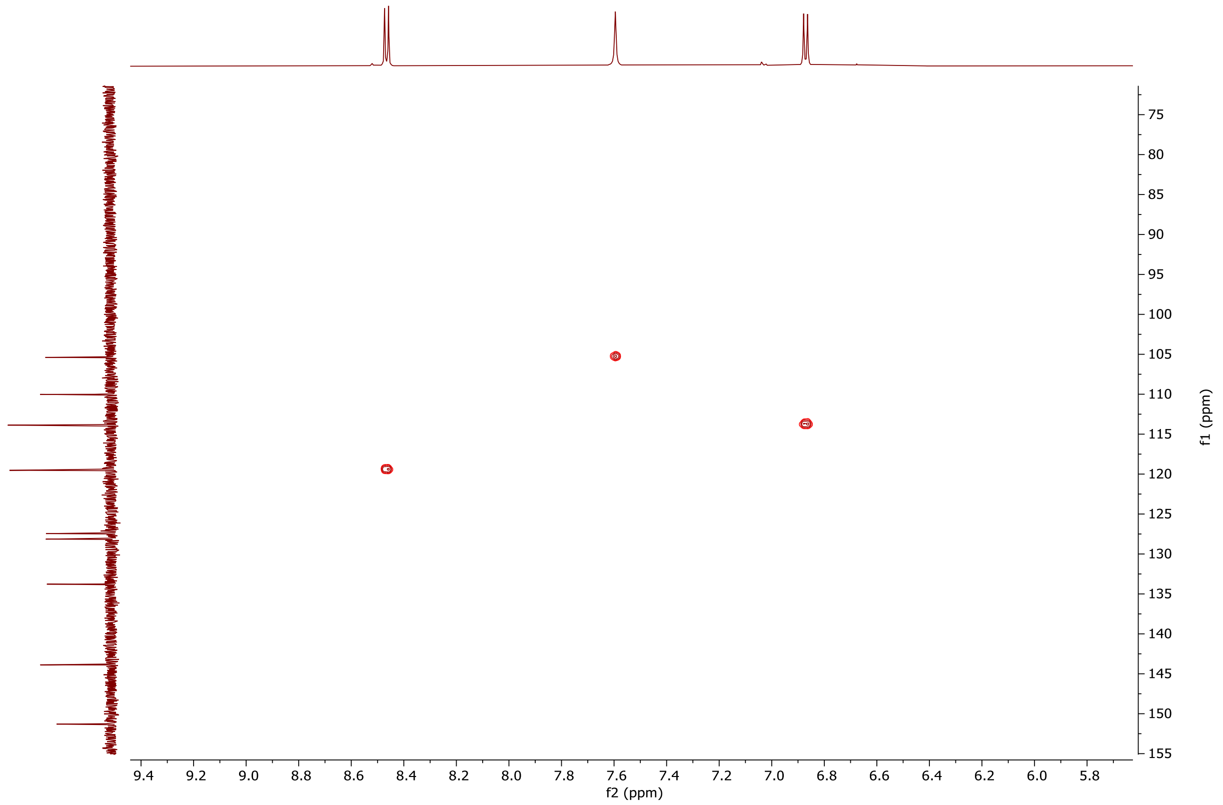

Figure S2-5. HSQC spectrum of compound **1** in MeOH-*d4*, 600/150 MHz

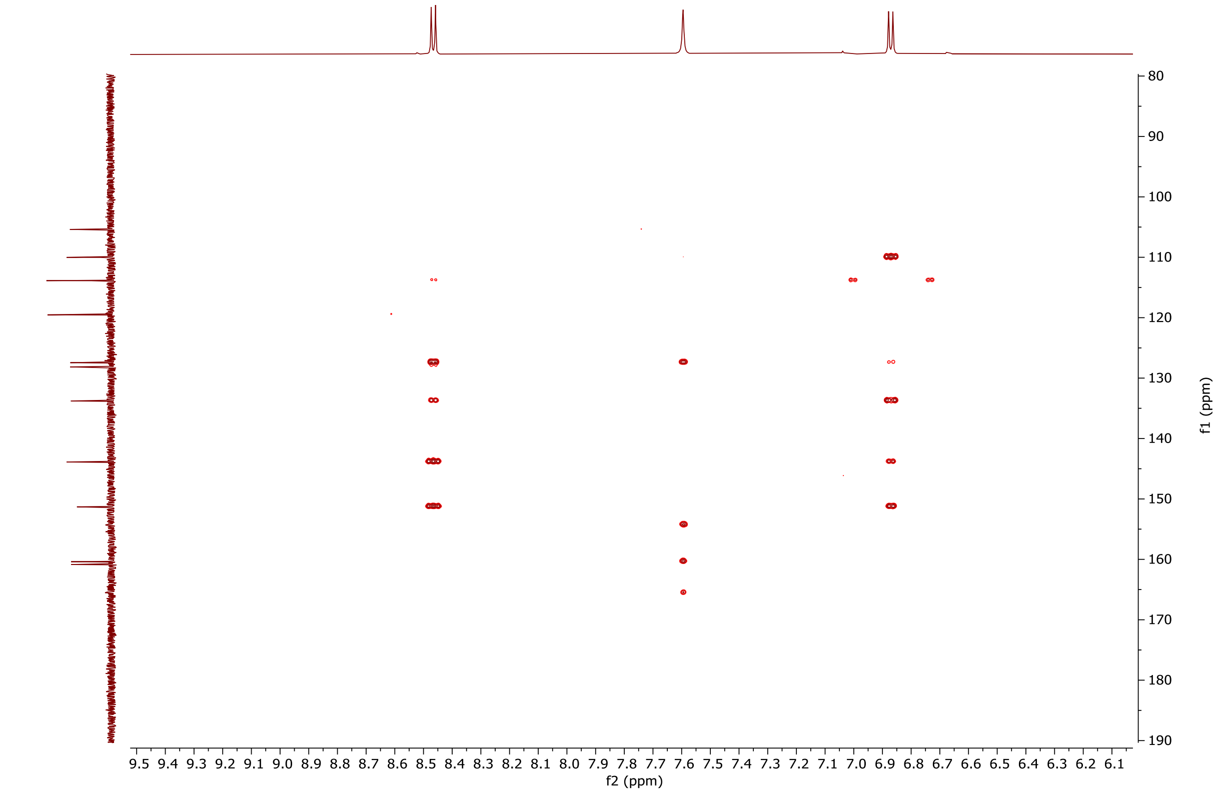

Figure S2-6. HMBC spectrum of compound **1** in MeOH-*d4,* 600/150 MHz

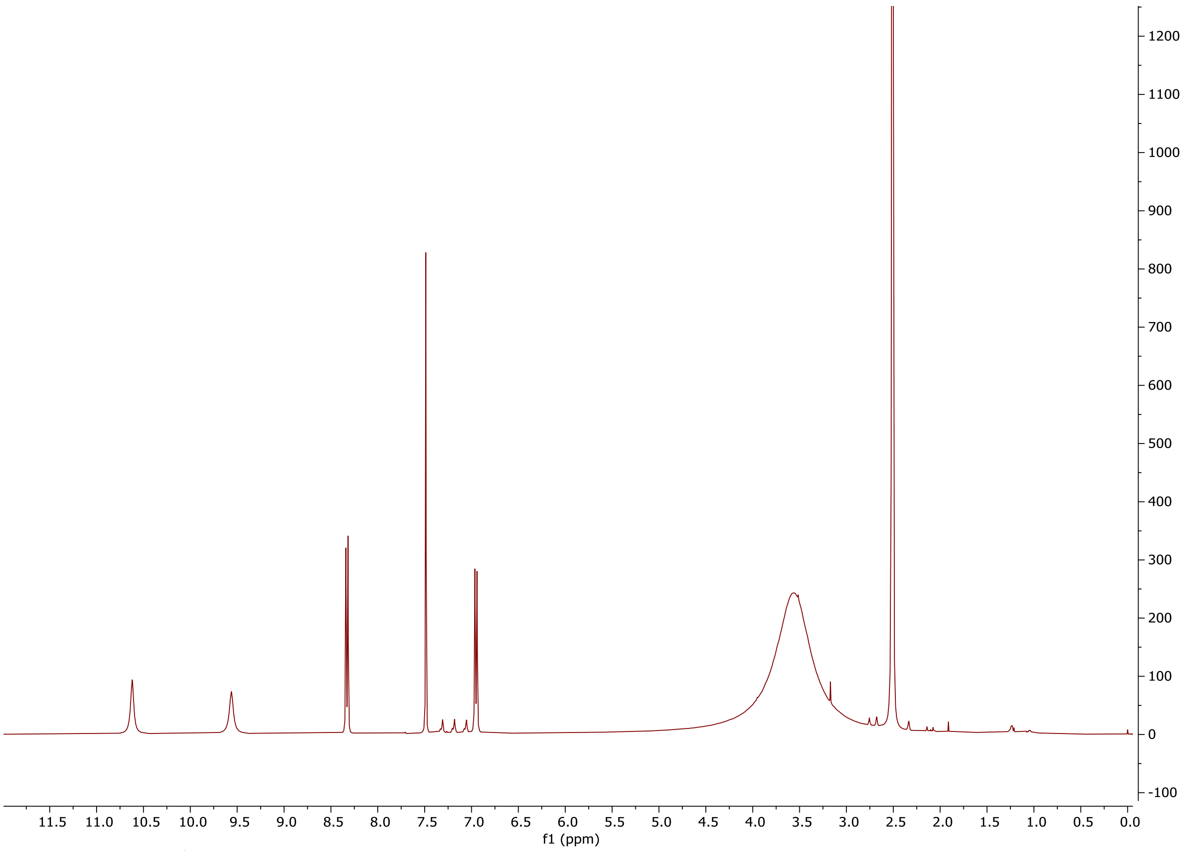

Figure S2-7. ^{1}H NMR spectrum of compound **1** in DMSO-*d6*, 400 MHz

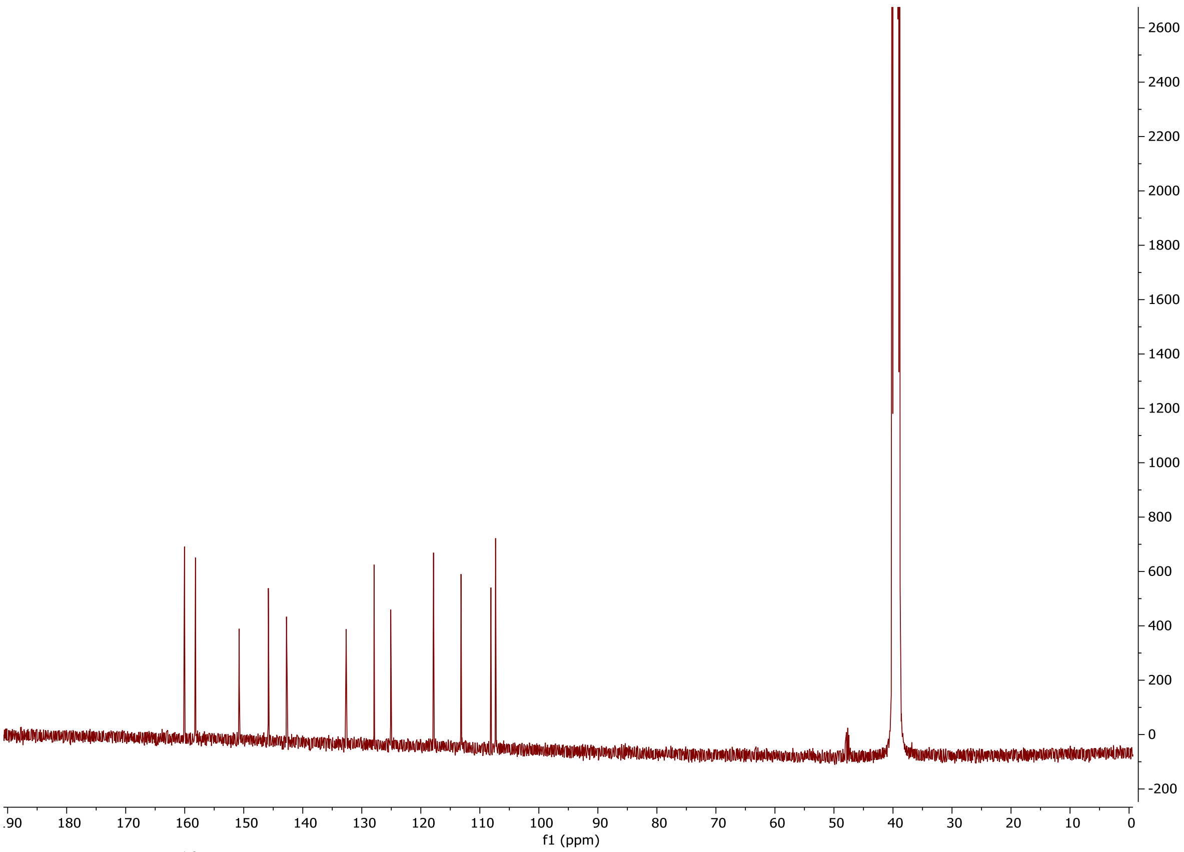

Figure S2-8. ^{13}C NMR spectrum of compound **1** in DMSO-*d6*, 100 MHz

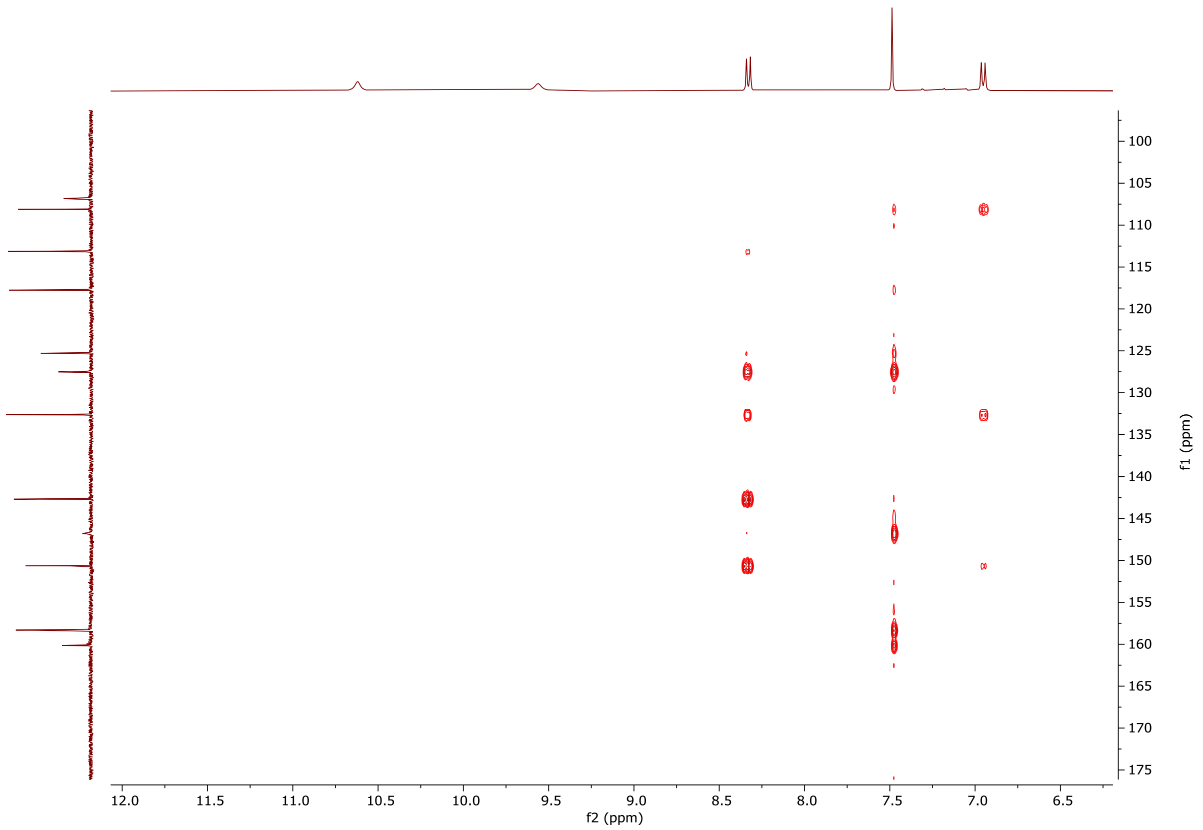

Figure S2-9. HMBC spectrum of compound **1** in DMSO-*d6*, 600/150 MHz

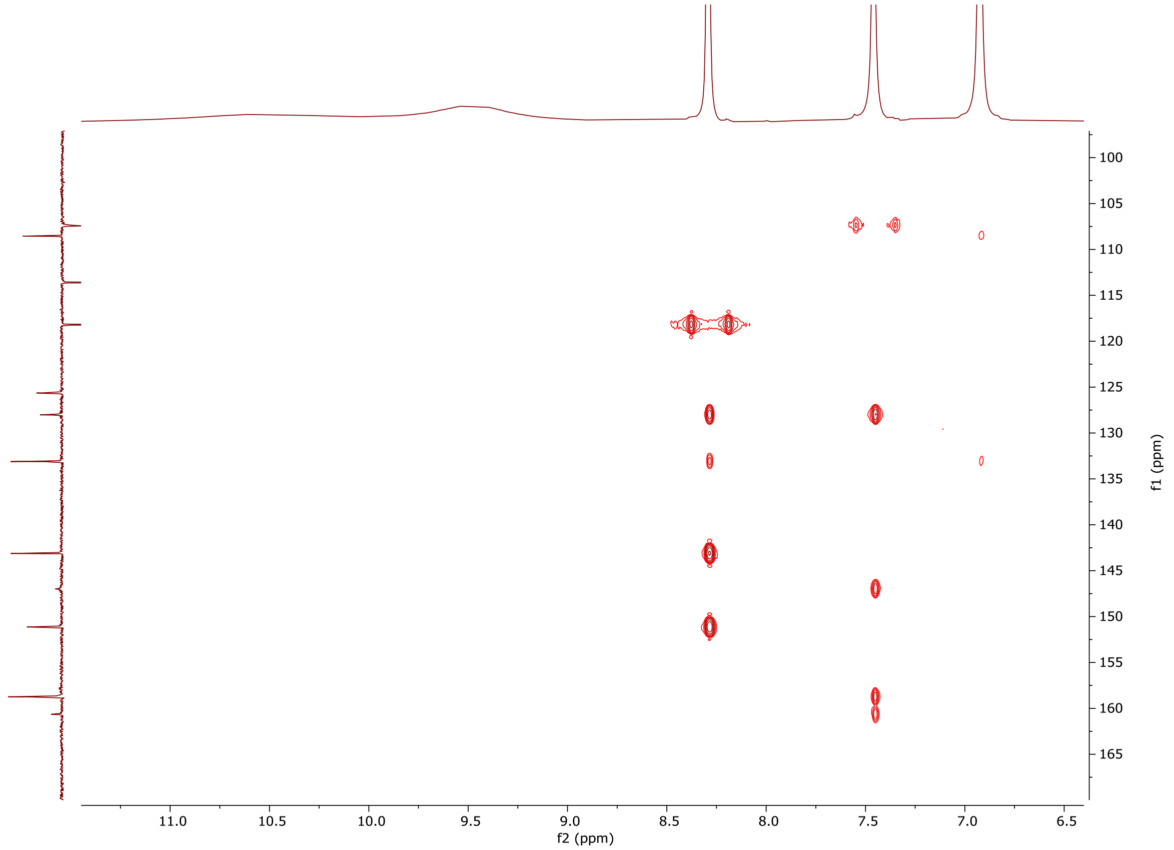

Figure S2-10. HMBC spectrum of compound **1** in DMSO-*d6*, 900/226 MHz

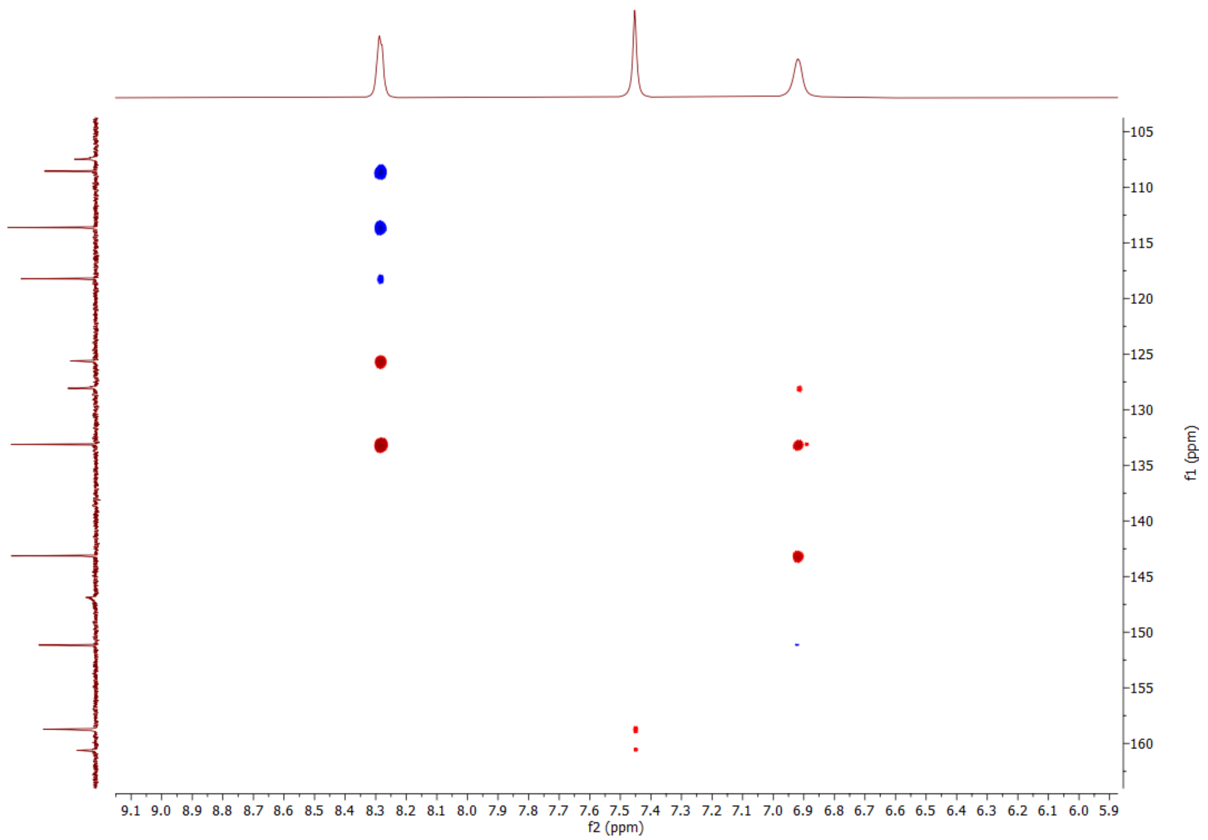

Figure S2-11. 1,n-ADEQUATE spectrum of compound **1** in DMSO-*d6*, 800/200 MHz

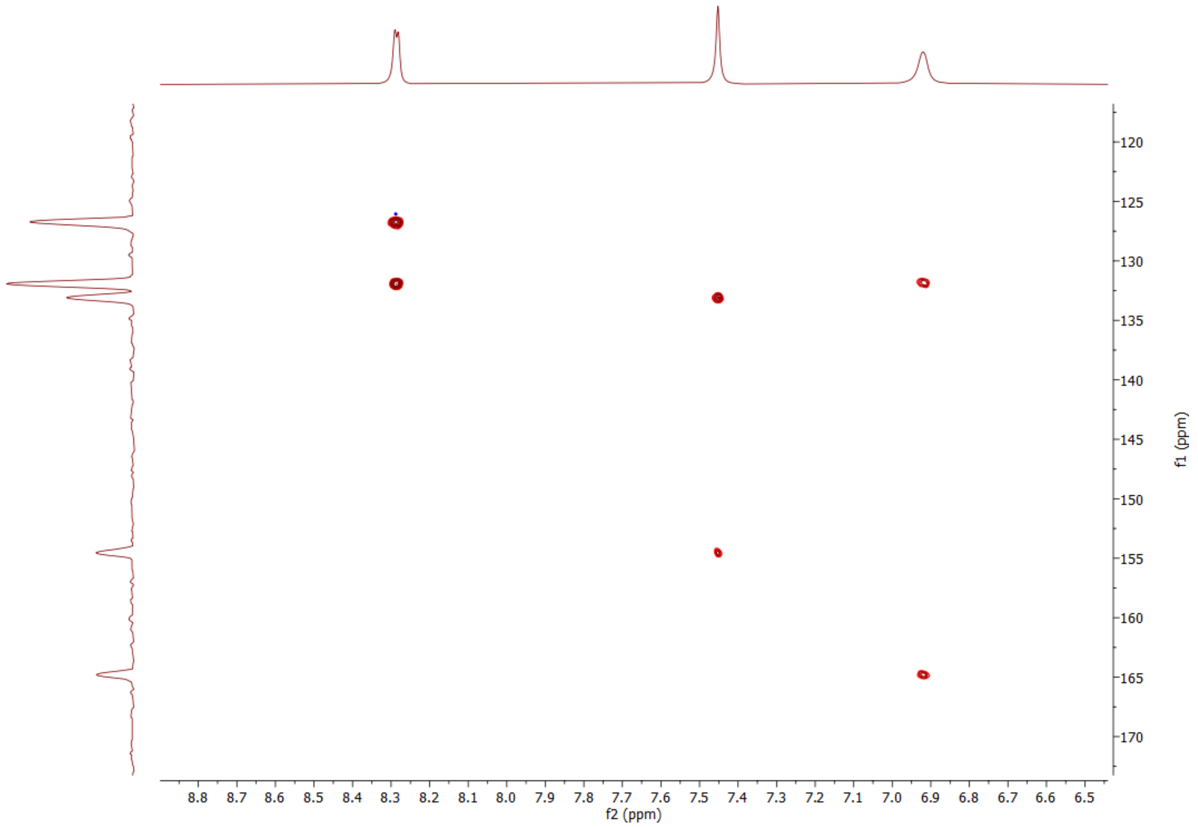

Figure S2-12. 1,1-ADEQUATE spectrum of compound **1** in DMSO-*d6*, 900/226 MHz

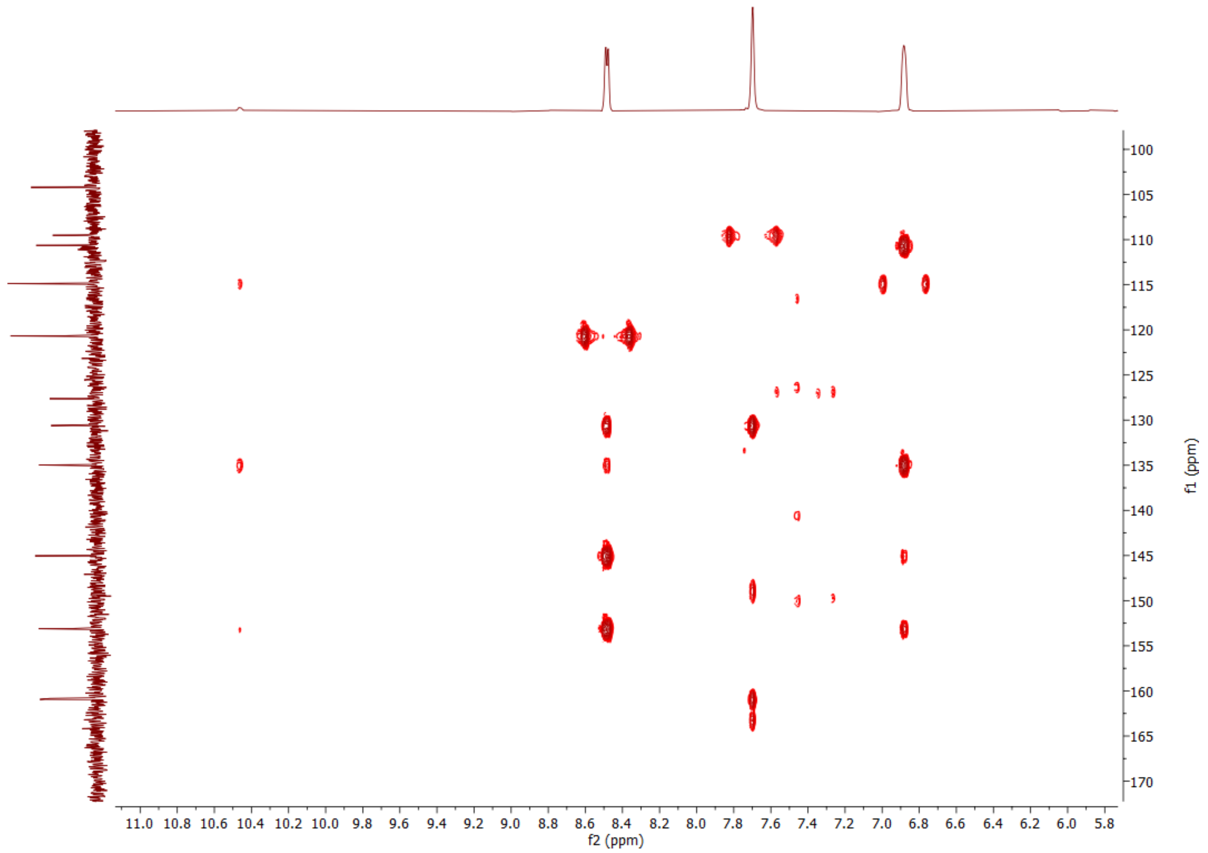

Figure S2-13. HMBC spectrum of compound **1** in MeOH-*d3*, 700/176 MHz, 253 K

Table S1-1. ^{1}H NMR data of compound **1** acquired in different solvents and under varying field strengths

**No.**	**type**	**δ$_H$[a] MeOH-*d4* 600 MHz**	**δ$_H$ MeOH-*d4* 400 MHz**	**δ$_H$ DMSO-*d6* 400 MHz**	**δ$_H$ DMSO-*d6* 800 MHz[c]**	**δ$_H$ DMSO-*d6* 900 MHz[c]**	**δ$_H$ DMSO-*d6* 600 MHz[c]**	**δ$_H$[b] MeOH-*d3* 800 MHz[c]**	**δ$_H$[b] MeOH-*d3* 700 MHz[c]**
4	CH	7.59, s	7.69, s	7.49, s	7.45, s	7.45, s	7.52, s	7.70, s	7.70, s
9	CH	6.87, d (9.0)	6.88, d (9.0)	6.95, d (9.0)	6.92, br d (6.8)	6.92, br d (7.8)	6.99, br d (8.3)	6.89, d (9.0)	6.88, d (9.0)
10	CH	8.46, d (9.0)	8.45, d (9.0)	8.33, d (9.0)	8.28, d (6.8)	8.28, d (7.8)	8.35, d (8.3)	8.48, d (9.0)	8.48, d (9.0)
7	COH			9.56, s	9.52, s	9.53, s	9.60, s	9.74, s	9.90, s
8	COH			10.62, s	10.60, s	10.65, s	10.70, s	10.30, s	10.46, s

[a] compound obtained under non-acidic conditions
[b] low temperature

Table S1-2. ^{13}C NMR data of compound **1** acquired in different solvents and under varying field strengths

**No.**	**type**	**δ$_C$[a] MeOH-*d4* 151 MHz**	**δ$_C$ MeOH-*d4* 126 MHz**	**δ$_C$ DMSO-*d6* 101 MHz**	**δ$_C$ DMSO-*d6* 201 MHz[c]**	**δ$_C$ DMSO-*d6* 151 MHz[c]**	**δ$_C$ DMSO-*d6* 226 MHz[c]**	**δ$_C$[b] MeOH-*d3* 176 MHz**
1	C=O	160.3	159.7	158.2	158.7	158.7	158.7	158.5
3	C	154.4[c]	147.4	145.8	146.9	146.9	147.9	144.7
4	CH	105.3	108.9	107.3	107.4	107.4	107.4	107.17
4a	C	128.1	126.5	125.1	125.6	125.6	125.7	125.2
5	C=O	160.8	159.9	158.3	158.7	158.7	158.7	158.6
6a	C	143.8	144.3	142.8	143.1	143.0	143.1	142.6
7	C	133.7	133.9	132.7	133.1	133.1	133.0	132.6
8	C	151.3	152.2	150.8	151.2	151.1	151.1	150.8
9	CH	113.8	114.1	113.2	113.6	113.6	113.6	112.6
10	CH	119.5	120.0	117.9	118.2	118.3	118.2	118.4
10a	C	110.0	109.7	108.1	108.5	108.5	108.5	108.3
10b	C	127.4	129.9	127.9	128.0	128.1	128.1	128.2
11	C=O	165.6[c]	161.8	160.0	160.7	160.5	160.6	161.8

[a] compound obtained under non-acidic conditions
[b] low temperature
[c] derived from HMBC

Table S1-3. HMBC data of compound **1** acquired in different solvents and under varying field strengths

**No.**	**CD_3OD-*d4*[a] (600, 151MHz)**	**DMSO-*d6* (600, 151 MHz)**	**CD_3OD-*d4*[a] (500, 126MHz)**	**DMSO-*d6* (600, 151 MHz)**	**CD_3OH-*d3*[b] (701, 176 MHz)**	**CD_3OH-*d3*[b] (800, 201 MHz)**	**DMSO-*d6* (900, 226 MHz)**
4	3, 5, 10a, 10b, 11	3, 4a, 5, 10a, 10b, 11	3, 5, 10b, 11	3, 5, 10b, 11	3, 5, 10b, 11	5, 10b, 11	3, 5, 10b, 11
9		6a, 7, 8, 10a	6a, 7, 8, 10a	-	6a, 7, 8, 10a	6a, 7, 8, 10, 10a, 10b	7, 10a
10		4a, 6a,7, 8, 9, 10b	6a, 7, 8, 10b	6a, 7, 8, 10b	6a, 7, 8, 10b	1, 4a, 6a, 7, 8, 9, 10b	6a, 7, 8, 10b
7-OH					-		
8-OH					7, 8, 9		

blue = weak signal
[a] compound obtained under non-acidic conditions
[b] low temperature

7,8-Dihydroxy-1,5-dioxo-1,5-dihydropyrano[4,3-c]chromene-3,10-dicarboxylic acid (**5**)

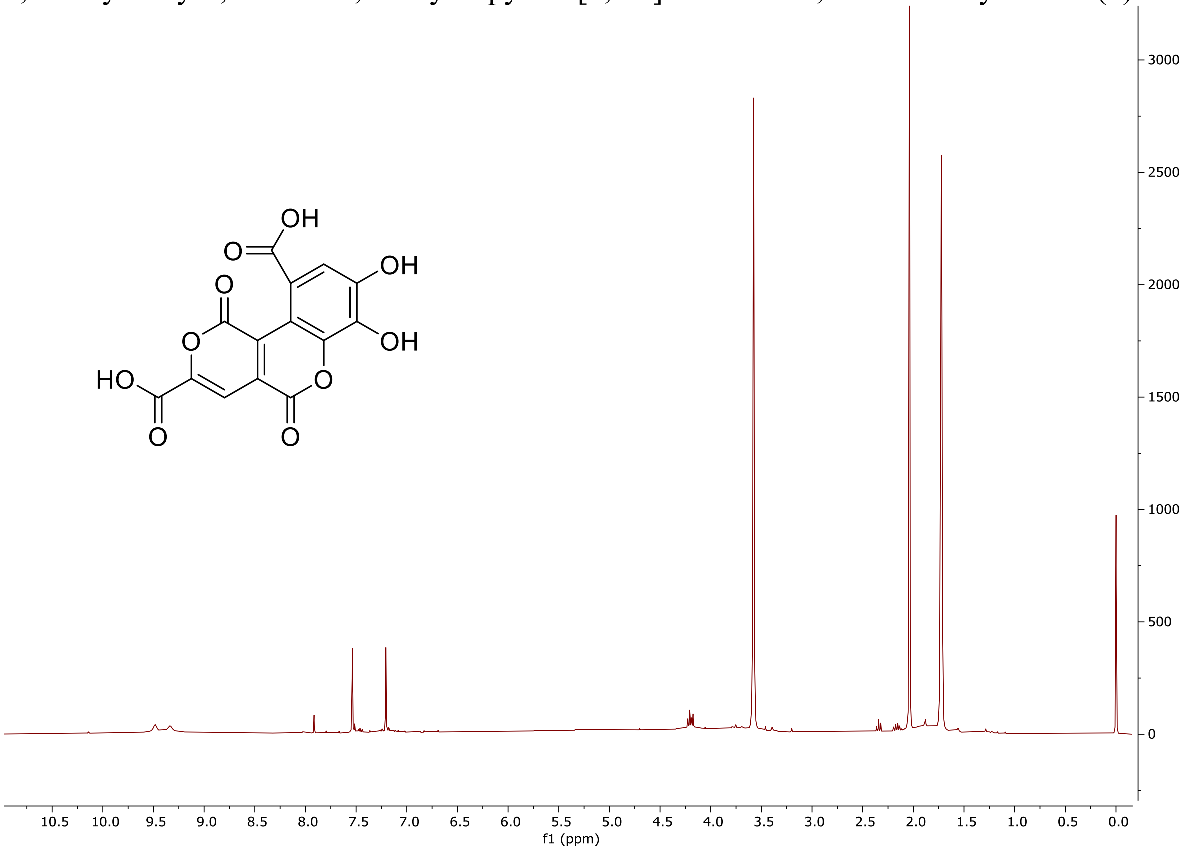

Figure S3-1. ^{1}H spectrum of compound **5** in THF-*d8*, 400 MHz

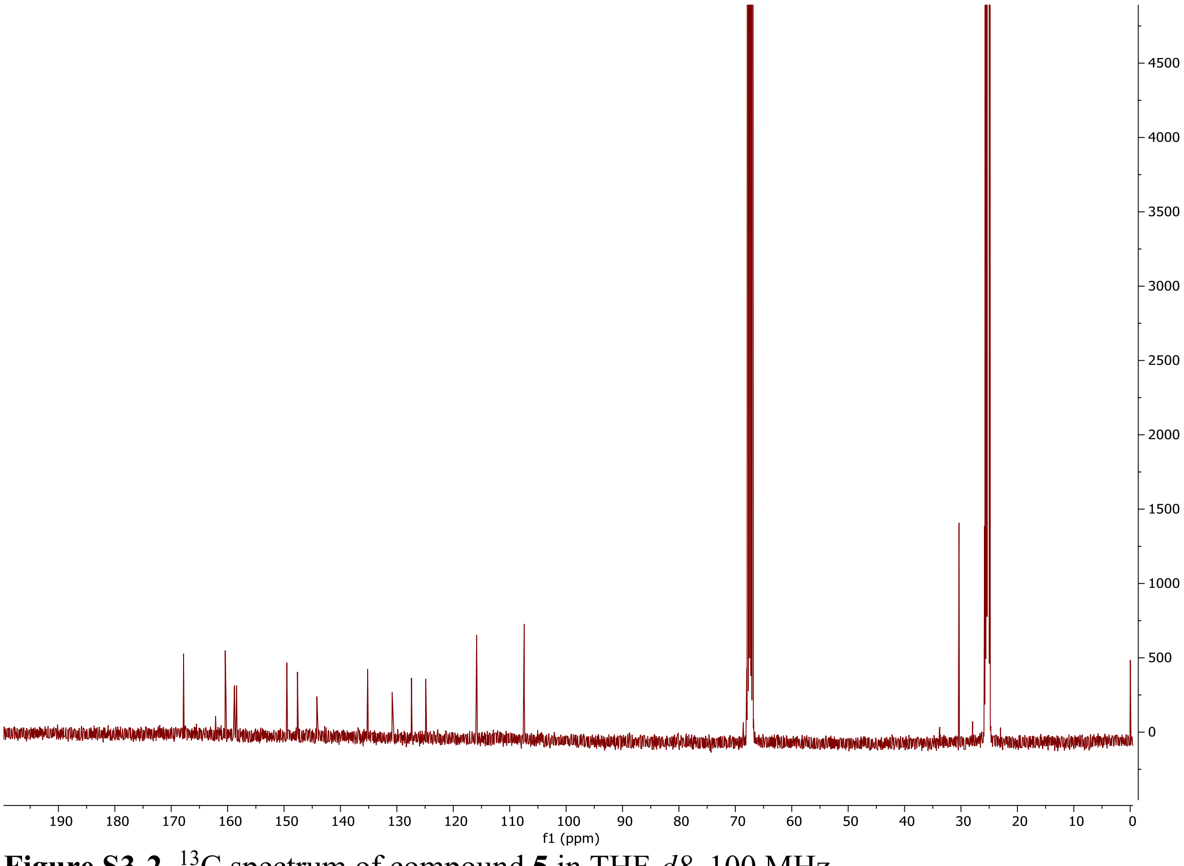

Figure S3-2. ^{13}C spectrum of compound **5** in THF-*d8*, 100 MHz

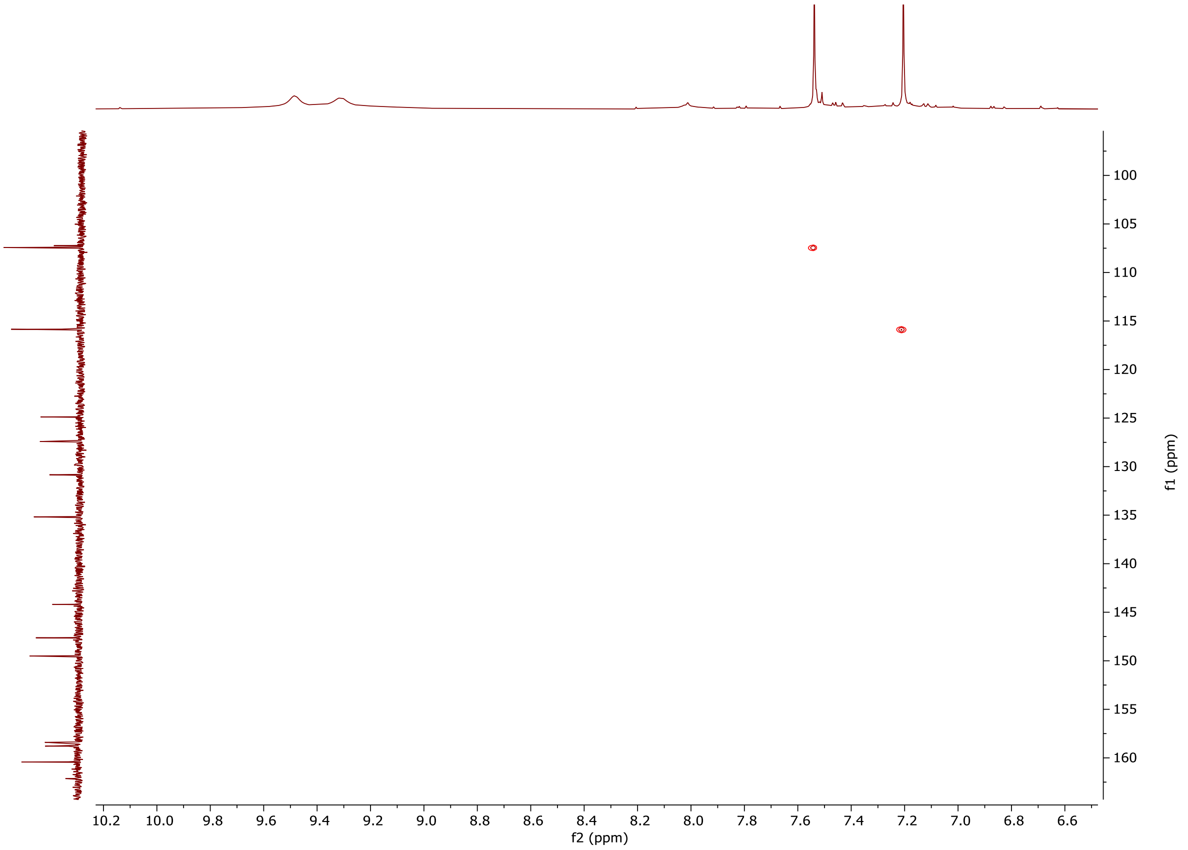

Figure S3-3. HSQC spectrum of compound **5** in THF-*d8*, 400/100 MHz

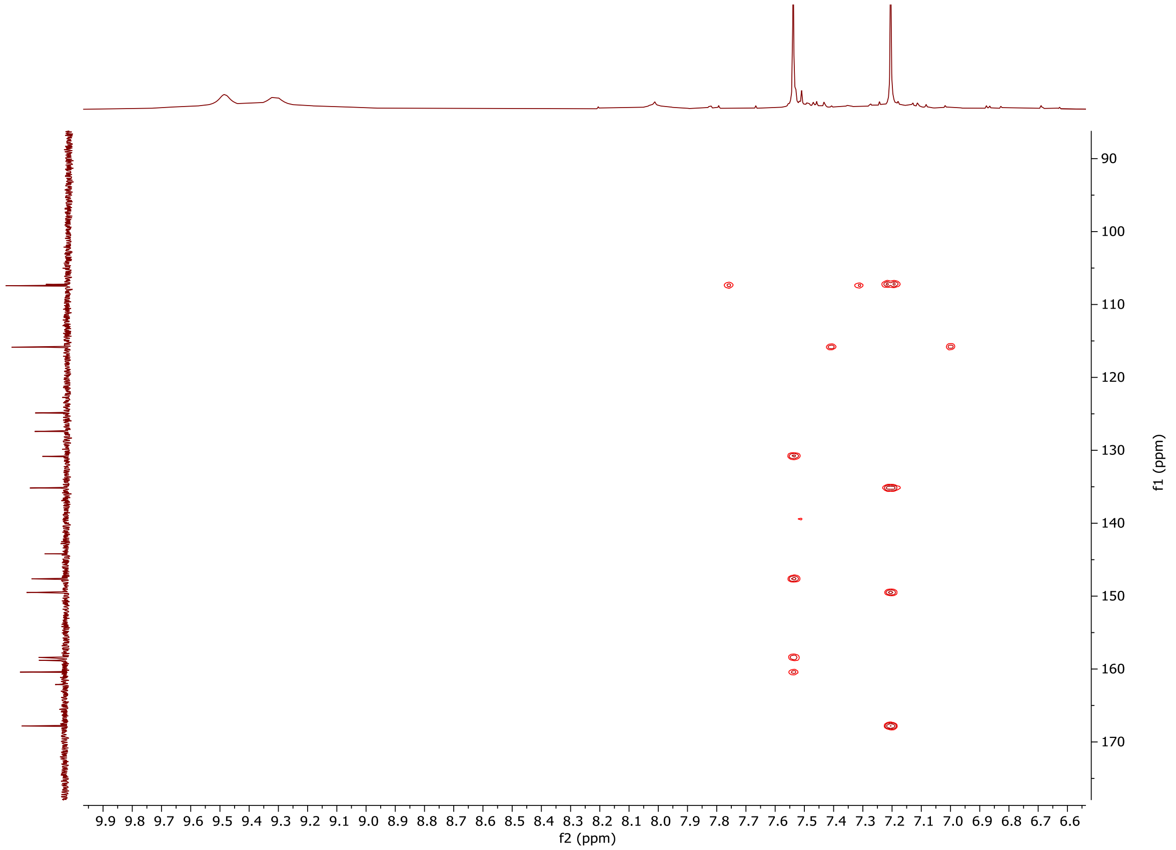

Figure S3-4. HMBC spectrum of compound **5** in THF-*d8*, 400/100 MHz

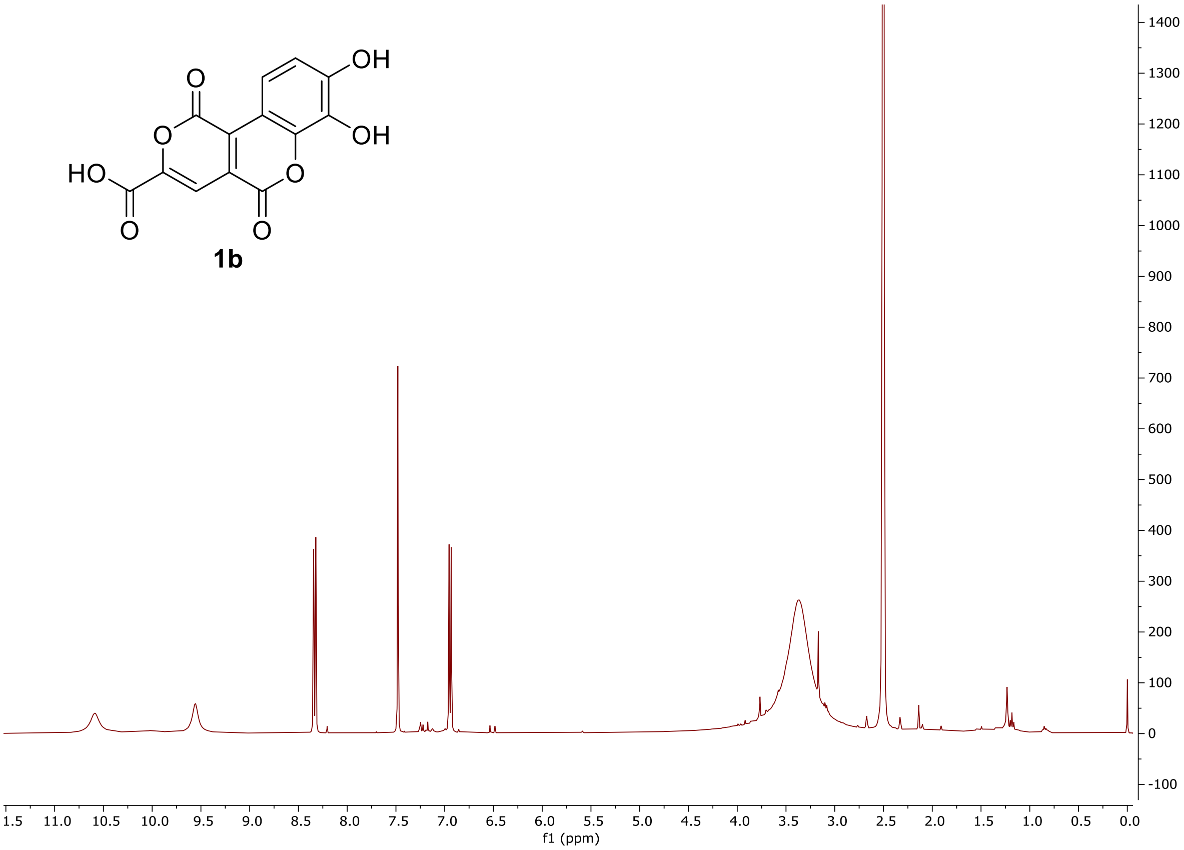

Figure S4-1. ^{1}H spectrum of compound **1b** in DMSO-*d6*, 400MHz

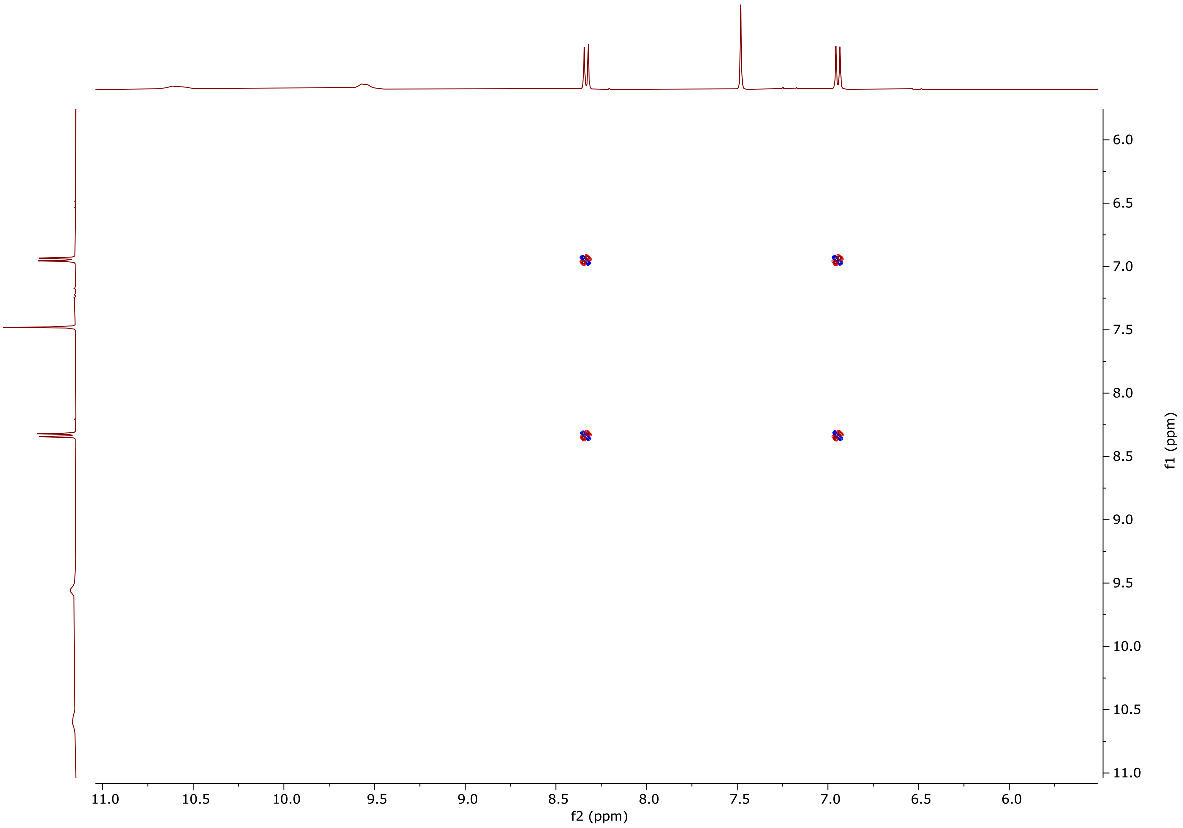

Figure S4-2. COSY spectrum of compound **1b** in DMSO-*d6*, 400MHz

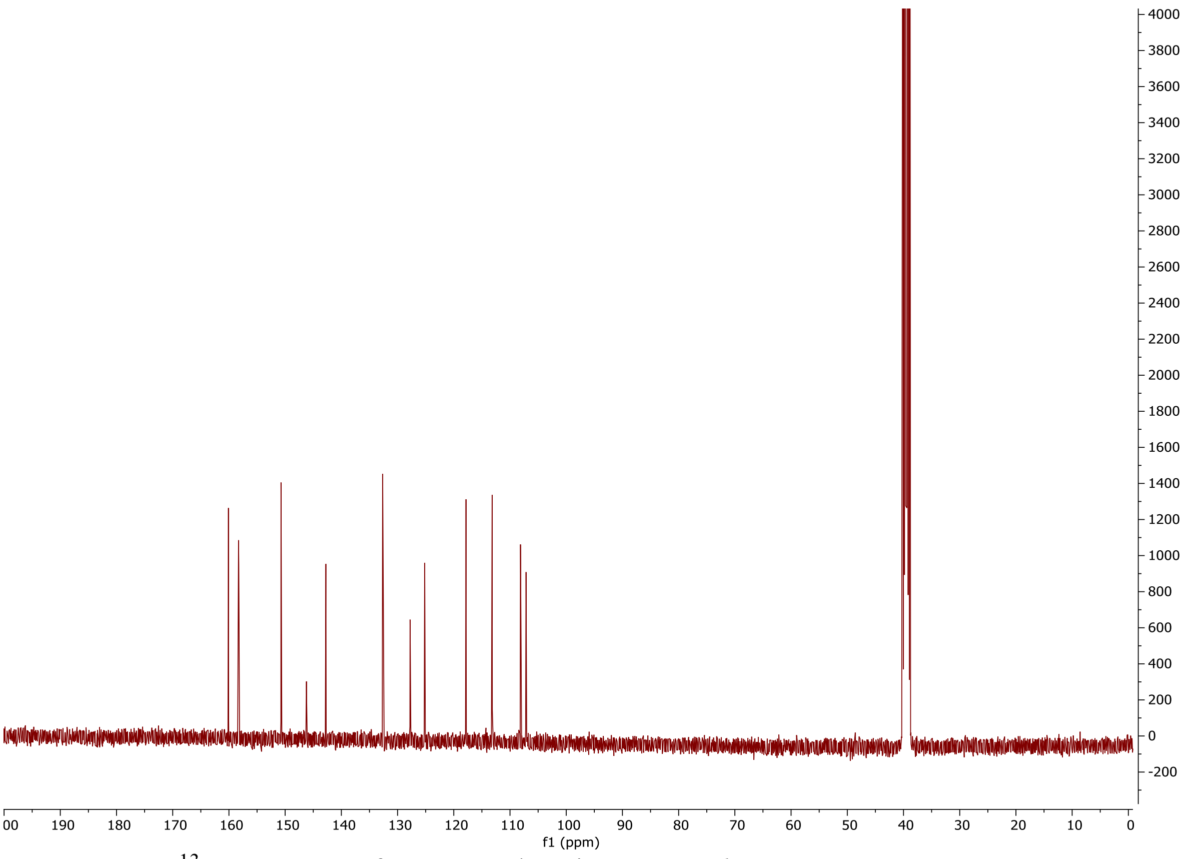

Figure S4-3. ^{13}C spectrum of compound **1b** in DMSO-*d6*, 100MHz

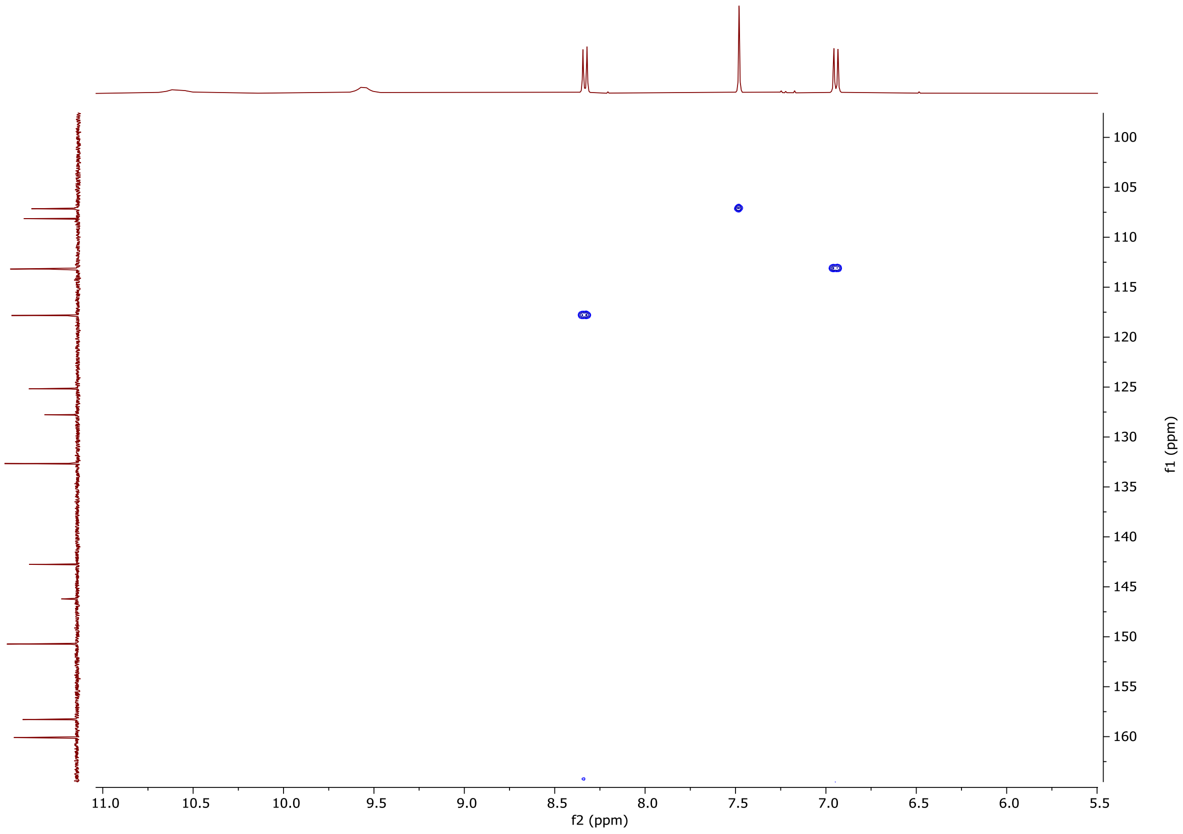

Figure S4-4. HSQC spectrum of compound **1b** in DMSO-*d6*, 400/100MHz

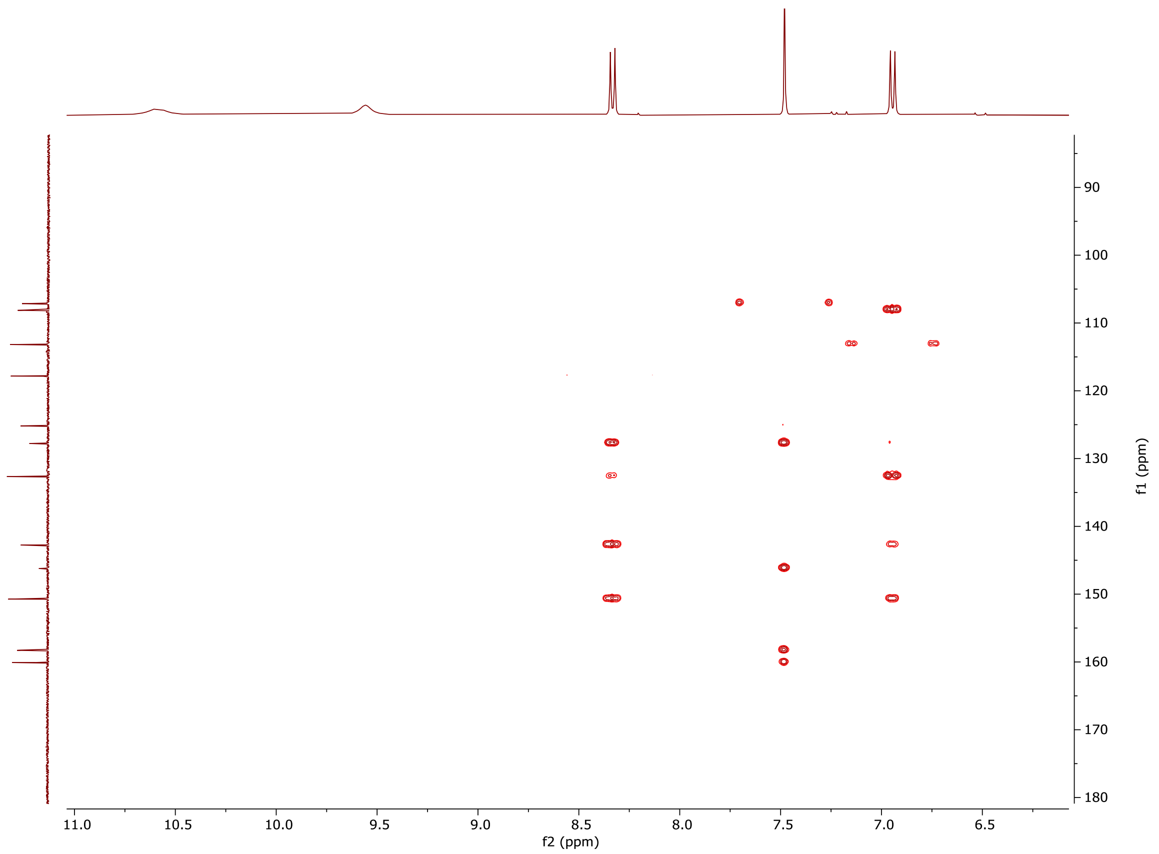

Figure S4-5. HMBC spectrum of compound **1b** in DMSO-*d6*, 400/100MHz

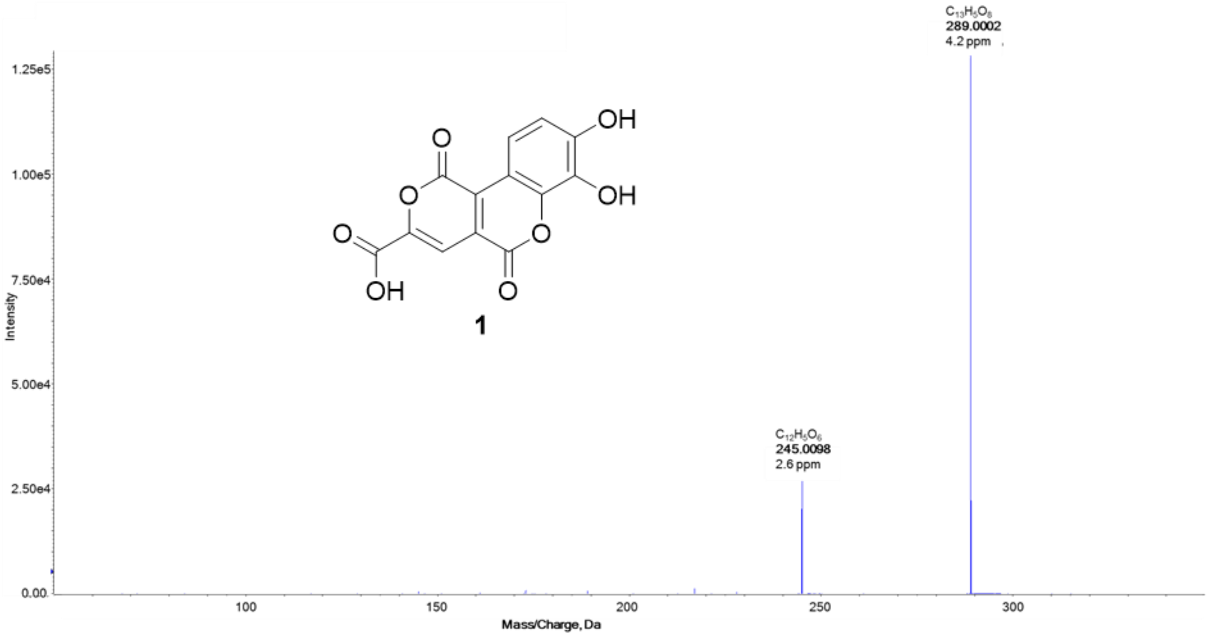

Figure S5-1. High resolution mass spectra (HRMS) acquired with quadrupole-time-of-flight-tandem instrument (QqTOF-MS) of compound **1.**

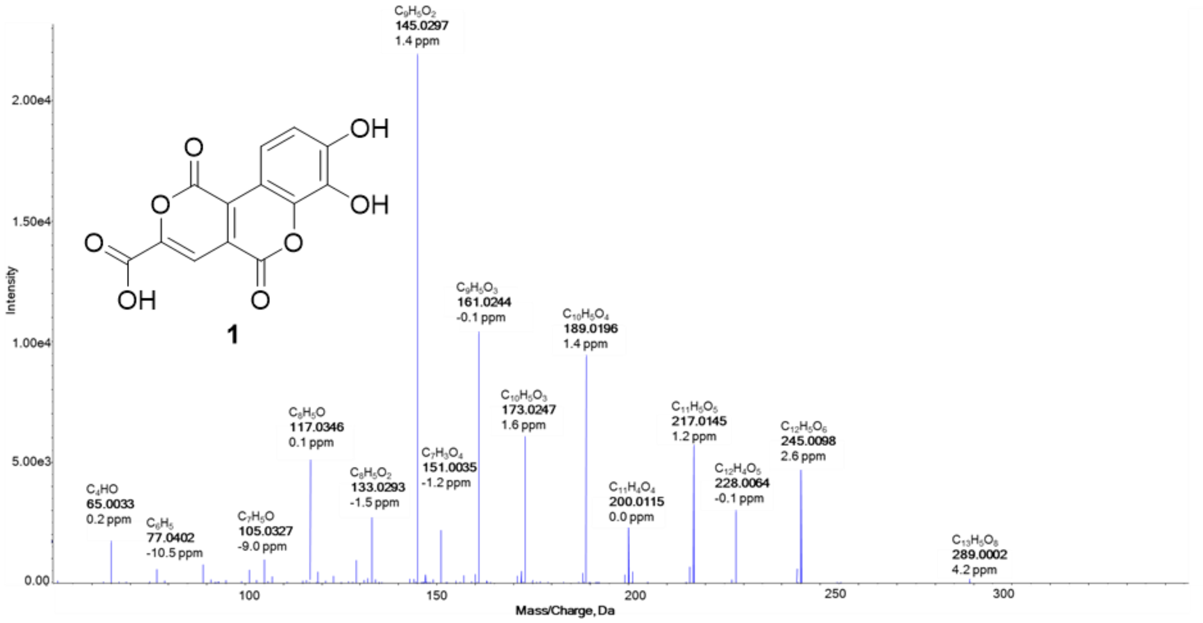

Figure S5-2. MS^2 of compound **1** acquired with quadrupole-time-of-flight-tandem instrument (QqTOF-MS/MS).

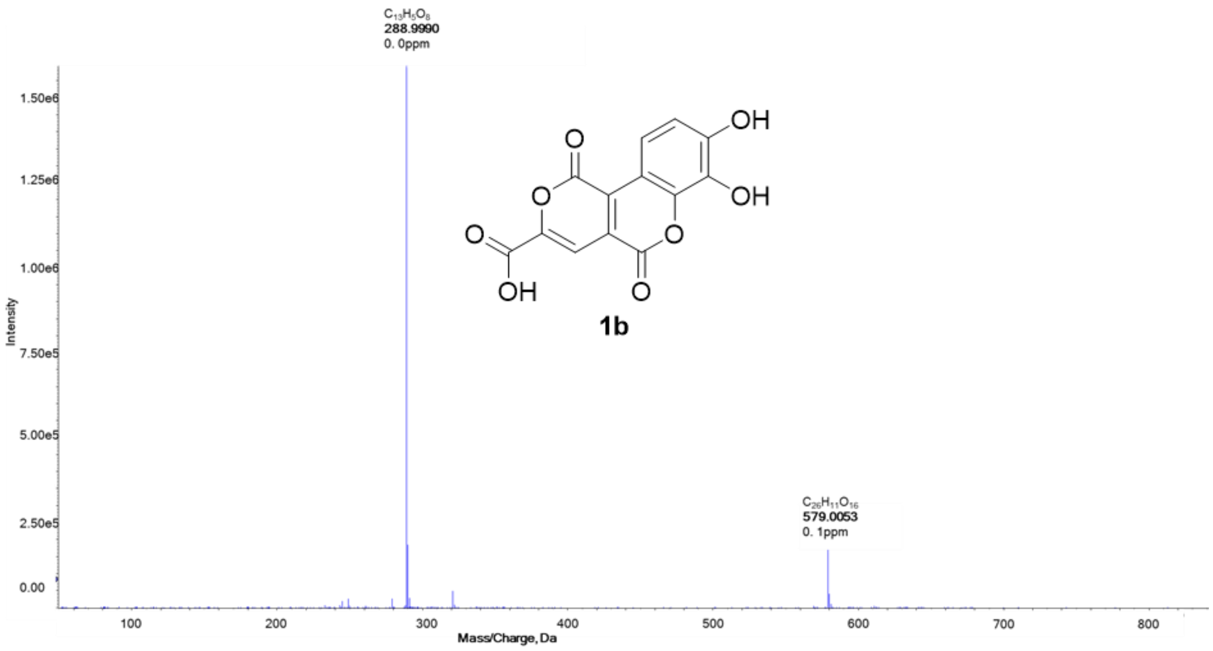

Figure S6-1. High resolution mass spectra (HRMS) acquired with quadrupole-time-of-flight-tandem instrument (QqTOF-MS) of synthetic compound **1b**

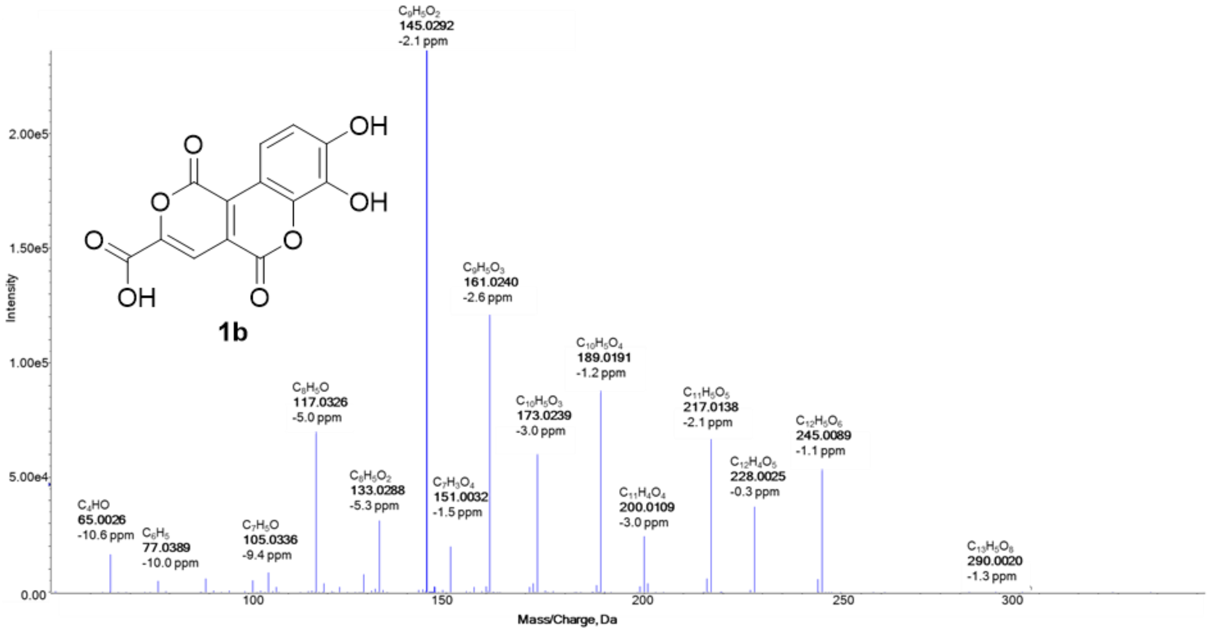

Figure S6-2. MS^2 of synthetic compound **1b** acquired with quadrupole-time-of-flight-tandem instrument (QqTOF-MS/MS)

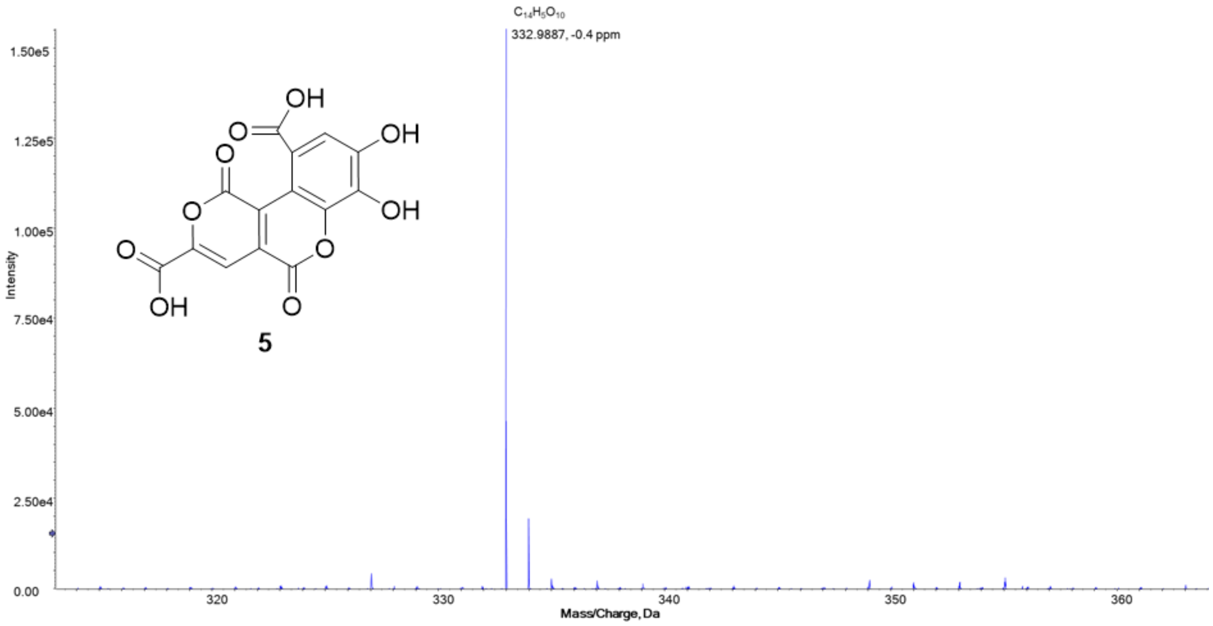

Figure S7-1. High resolution mass spectra (HRMS) acquired with quadrupole-time-of-flight-tandem instrument (QqTOF-MS) of synthetic compound **5**

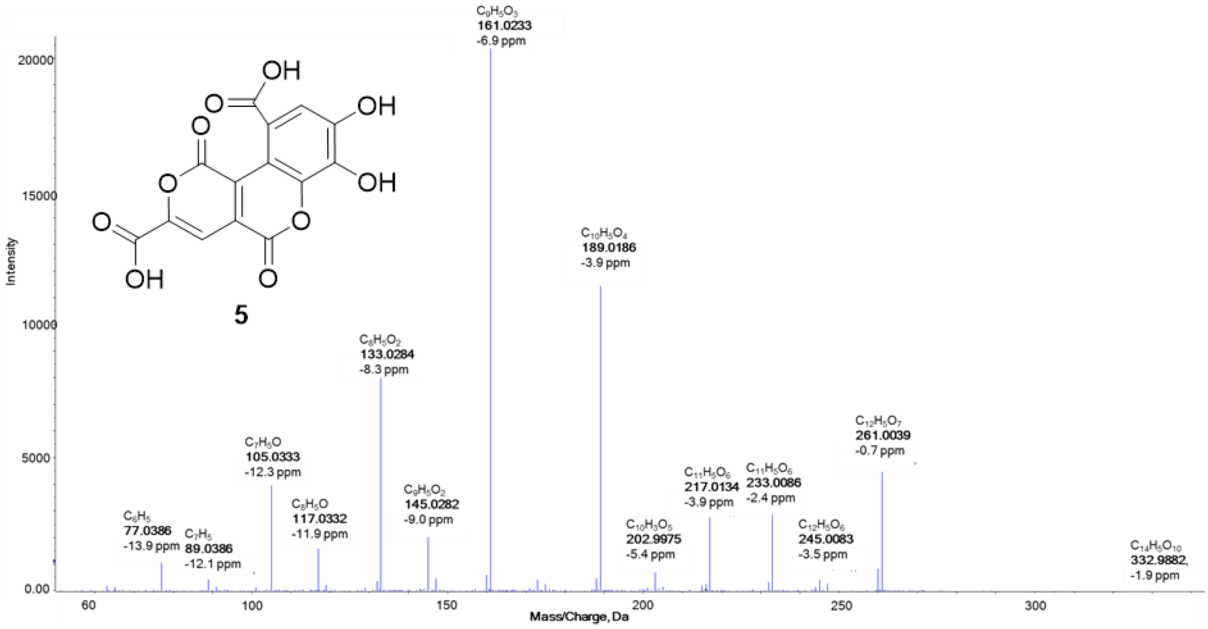

Figure S7-2. MS^2 of synthetic compound **5** acquired with quadrupole-time-of-flight-tandem instrument (QqTOF-MS/MS)

Table S2-1. DFT calculations: comparison between experimental and calculated chemical shifts*

Molecular structure and calculated *J*-coupling	δ(^{1}H), ppm	δ(^{13}C), ppm
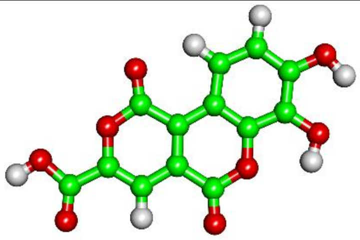 Lumnitzeralactone (**1**), $^3J_{HH}$ = 8.1 Hz	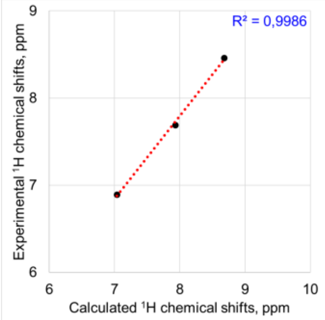	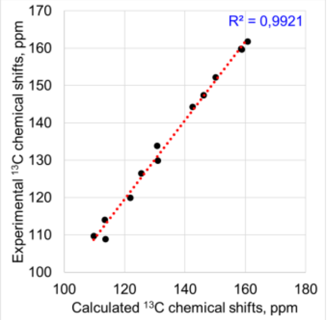
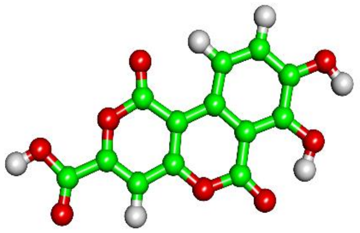 Isomer (**II**), $^3J_{HH}$ = 7.7 Hz	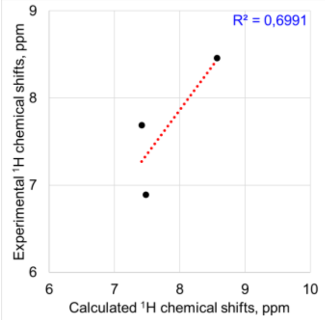	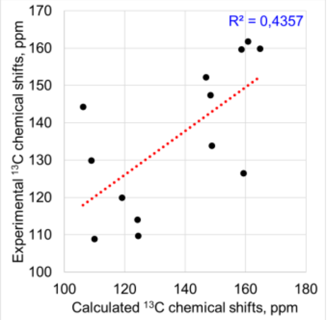
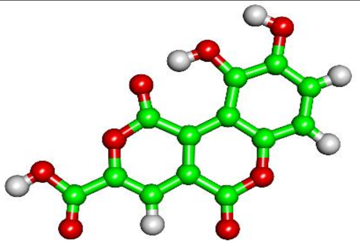 Isomer (**III**), $^3J_{HH}$ = 7.5 Hz	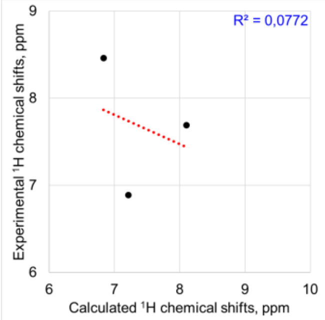	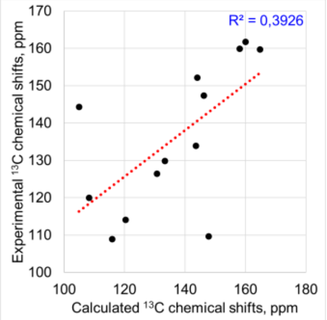
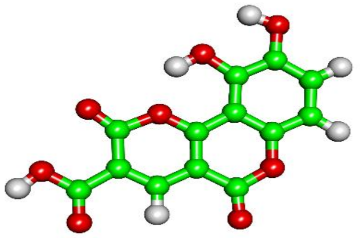 Isomer (**IV**), $^3J_{HH}$ = 7.7 Hz	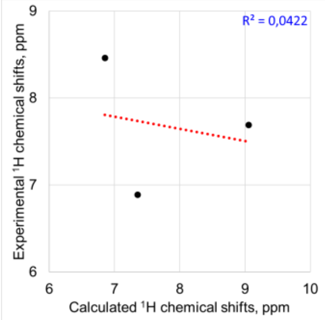	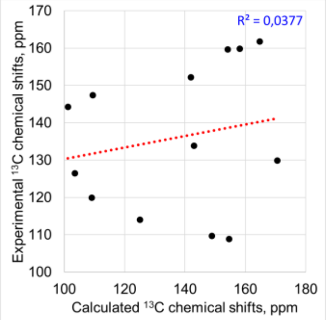
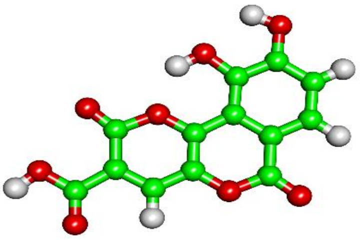 Isomer (**V**),$^3J_{HH}$ = 7.4 Hz	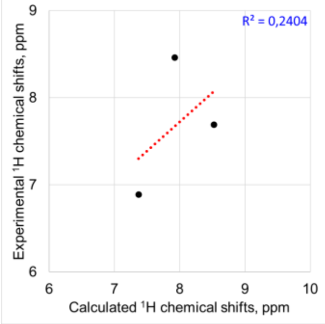	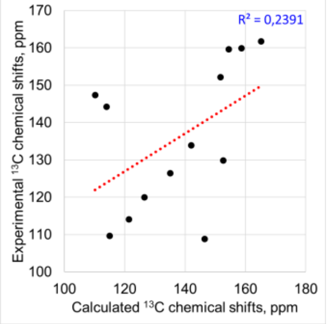

* Geometry optimization: # opt b3lyp/6-31+g(d,p) geom=connectivity;
NMR: # nmr=(giao,spinspin) mpw1pw91/6-311+g(2d,p) scrf=(iefpcm,solvent=methanol) geom=connectivity

Table S2-2. Cartesian coordinates of structures A-E used in DFT calculations

Structure	Cartesian Coordinates			
Lumnitzeralactone (**1**)	**Element**	**X**	**Y**	**Z**
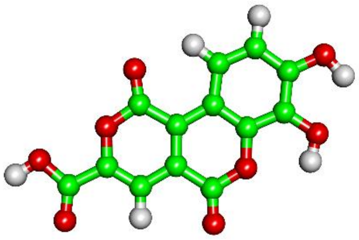	C	-1.94132100	0.54152900	0.00026300
	C	-3.33084100	0.50949900	-0.00030500
	C	-3.98314000	-0.72941700	-0.00082200
	C	-3.22924500	-1.91141900	-0.00050000
	C	-1.84330900	-1.86882800	0.00018300
	C	-1.15460200	-0.63107300	0.00053400
	C	0.27884300	-0.44001400	0.00075300
	C	0.81984100	0.83835500	0.00065600
	C	-0.04131300	2.03720300	0.00080200
	O	-1.40116600	1.80630300	0.00049700
	C	1.21064600	-1.59411500	0.00133900
	O	2.56367800	-1.29172600	-0.00003900
	C	3.05078900	-0.02352500	-0.00016200
	C	2.23161200	1.05309500	0.00033500
	O	-5.33558800	-0.79300300	-0.00152400
	O	-4.10945400	1.63388700	-0.00089900
	O	0.34751100	3.18120500	0.00127700
	O	0.90621200	-2.76447900	0.00248000
	C	4.53371200	0.09503900	-0.00100800
	O	5.10957000	1.16480800	-0.00101700
	O	5.15801700	-1.09716300	-0.00189500
	H	-3.75590200	-2.85933000	-0.00075300
	H	-1.27151300	-2.78523200	0.00050300
	H	2.64049200	2.05464800	0.00035300
	H	-5.69923700	0.10672600	-0.00152600
	H	-3.54784100	2.42413900	0.00043200
	H	6.11372000	-0.91758000	-0.00246000
Isomer (**II**)	**Element**	**X**	**Y**	**Z**
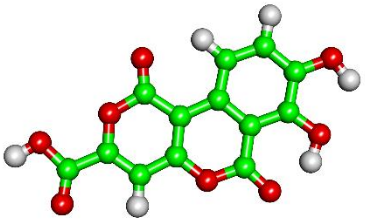	C	-1.86489300	0.51052000	0.00016300
	C	-3.26876200	0.39705300	0.00012100
	C	-3.86340200	-0.87719600	0.00002900
	C	-3.05494400	-2.01153900	0.00006300
	C	-1.66225800	-1.91267800	0.00003800
	C	-1.03947400	-0.65605600	0.00007800
	C	0.39892900	-0.43641100	-0.00002300
	C	0.88959500	0.85337000	-0.00016900
	C	1.36759100	-1.53803600	-0.00014000
	O	2.72307600	-1.17418000	-0.00017500
	C	3.14446900	0.10719700	-0.00033200
	C	2.28020800	1.15064100	-0.00032400
	O	-5.21409500	-1.00004200	-0.00000600
	O	-4.11371000	1.45165500	0.00001300
	O	1.12821500	-2.72306400	-0.00013900
	C	4.62198300	0.30780700	-0.00048900
	O	5.13414900	1.40930600	0.00015100
	O	5.30901100	-0.84686500	0.00063400
	H	-3.53482400	-2.98491000	0.00004000
	H	-1.05658700	-2.80735700	0.00002700
	H	2.63989800	2.17086300	-0.00043100

H	-5.60848500	-0.11287900	-0.00022200
H	-3.56998600	2.27649100	-0.00042200
H	6.25435500	-0.61850900	0.00122200
C	-1.29358800	1.84276300	0.00026200
O	-1.92801500	2.89048300	0.00010400
O	0.07923300	1.94916900	-0.00006600

Isomer (**III**)

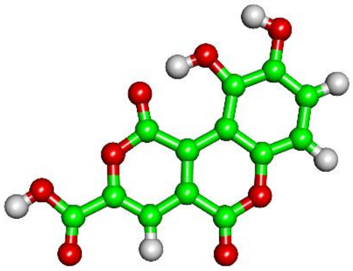

**Element**	**X**	**Y**	**Z**
C	1.93955700	1.36213900	0.01368000
C	3.28940800	1.66851600	0.01450300
C	4.22531900	0.63676100	0.00148800
C	3.79249200	-0.68168300	-0.01446000
C	2.41870100	-1.00490500	-0.01467300
C	1.42850800	0.02759200	0.00363000
C	-0.03188100	-0.09655300	0.00694600
C	-0.82525800	1.04795600	-0.00096100
C	-0.23734700	2.40570800	0.00213600
O	1.11508300	2.46861100	0.02124600
C	-0.76220400	-1.38448200	0.03411400
O	-2.12483300	-1.35064800	0.01567500
C	-2.85915100	-0.20687200	-0.00420700
C	-2.25005900	0.99768700	-0.00911900
O	-0.88686300	3.42825400	-0.00531600
O	-0.28038000	-2.51085200	0.07763400
C	-4.33477200	-0.38833000	-0.01670000
O	-5.10840300	0.54663200	-0.03395200
O	-4.70488900	-1.68159700	-0.00695800
H	-2.82670100	1.91308400	-0.02024400
H	-5.67722000	-1.70203700	-0.01635600
O	4.70775400	-1.68750200	-0.03206100
O	2.20852500	-2.33576600	-0.04319900
H	1.23416700	-2.55134400	0.00735000
H	4.22387600	-2.53015100	-0.03832600
H	5.29013900	0.84222400	0.00131000
H	3.58791300	2.70995900	0.02346300

Isomer (**IV**)

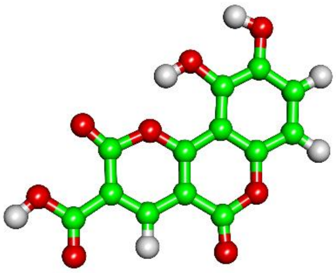

**Element**	**X**	**Y**	**Z**
C	-2.10813900	1.32765400	-0.00003200
C	-3.48682900	1.48435800	-0.00042300
C	-4.30032400	0.34949400	-0.00054600
C	-3.75196900	-0.93391800	-0.00016000
C	-2.36106200	-1.09914700	0.00002100
C	-1.51677100	0.03884800	0.00009000
C	-0.08668000	0.00095600	0.00016600
C	0.68010100	1.15115000	0.00057400
C	0.03830200	2.46991500	0.00062800
O	-1.34589100	2.46896700	0.00042000
C	2.72158200	-0.19851800	0.00037400
C	2.09082600	1.02296700	0.00034200
O	0.61538800	3.53112100	-0.00070200
C	4.21304300	-0.19512300	-0.00034000

O	4.87016800	0.82947300	0.00055400
O	4.77017500	-1.42001500	-0.00213000
H	2.69882300	1.92268100	0.00011500
H	5.73360000	-1.28612900	-0.00247900
O	-4.57721600	-2.01634100	-0.00029700
O	-1.92812600	-2.38737900	0.00003200
H	-0.95610000	-2.43155200	0.00025100
H	-4.03859300	-2.82305900	0.00016500
H	-5.38092000	0.44600000	-0.00141800
H	-3.90899900	2.48221800	-0.00007300
C	1.94597400	-1.42796400	0.00127500
O	2.26644200	-2.58162600	0.00105700
O	0.50204300	-1.20597100	0.00002000

Isomer (**V**)

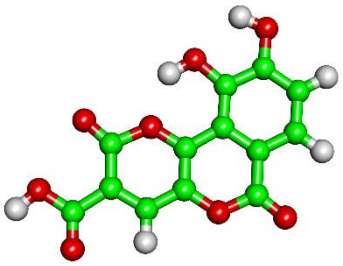

**Element**	**X**	**Y**	**Z**
C	-2.09352600	1.19109600	-0.00033300
C	-3.48950100	1.23289000	-0.00010700
C	-4.22842400	0.05252800	-0.00005900
C	-3.57970600	-1.18466700	0.00001000
C	-2.17347400	-1.25306800	-0.00009500
C	-1.42107900	-0.06509900	-0.00031000
C	0.01358000	-0.02061800	-0.00017400
C	0.69415000	1.16887500	-0.00009900
C	2.81962200	-0.01396300	-0.00008900
C	2.10331000	1.16205500	-0.00012600
C	4.30565600	0.10757900	-0.00016600
O	4.87794700	1.18318000	0.00109700
O	4.95987800	-1.06680100	-0.00182000
H	2.64411500	2.10373000	-0.00014600
H	5.90933200	-0.85536500	-0.00177500
O	-4.31070200	-2.32243800	0.00008900
O	-1.66228200	-2.51458900	-0.00032400
H	-0.68893900	-2.49805200	-0.00018200
H	-3.70955100	-3.08526900	0.00034000
H	-5.31296800	0.06769200	-0.00042600
H	-3.98181500	2.19840900	0.00025800
C	2.13657900	-1.30061400	0.00029700
O	2.55983100	-2.42462800	0.00191500
O	0.69824800	-1.18880800	-0.00004200
C	-1.34604800	2.46083900	-0.00027900
O	-1.83221100	3.56614100	0.00066800
O	0.04591400	2.37317500	-0.00019600

Structure Elucidation Report for "SE_Lumlac_aftercalculation" (Page 1)

Structure Elucidation Report for "SE_Lumlac_aftercalculation"

Initial Data

Composition Restrictions:
Molecular Weight = 0.000-1000.000
Double Bonds Equivalent = 0.00-100.00
Allowed Composition = C(0-100) H(0-100) O(0-20) N(0-10)
Molecular Formula = C13H6O8

Spectral Data:
standard 1H (user) - 5 peaks
merged 1H - 4 peaks
standard 13C (user) - 13 peaks
merged 13C - 13 peaks
COSY 1H-1H (user) - 2 peaks
HSQC 13C-1H (user) - 3 peaks
HMBC 13C-1H (user) - 20 peaks
1,n-ADEQUATE(inverted) 13C-1H (user) - 7 peaks
1,1-ADEQUATE 13C-1H (user) - 6 peaks

Result of Automatic Elucidation

No molecule(s) have been found by NMR spectra in 0 database(s).
0 stereoisomer(s) have been excluded from the search result.
No molecule(s) have been found by NMR spectra in 0 database(s).
0 stereoisomer(s) have been excluded from the search result.
1 Molecular Connectivity Diagram (MCD) has been created from 1 MF
Current Molecular Connectivity Diagram (MCD) passed all tests
No updates performed.
31061041 molecule(s) have been generated by Correlation Spectroscopy Based Generator and 44 molecule(s) have been stored.
Generation time: 18 h 10 m 27 s (Check: 0 s, Generation: 18 h 10 m 27 s 325 ms)
No (from No) connectivities have been extended during generation
ACD/CNMR Spectrum (Neural Net) has been calculated for 44 of 44 structure(s) from Generated Molecules
44 of 44 structure(s) have been stored in Generated Molecules after removing duplicates
ACD/HNMR Spectrum (Neural Net) has been calculated for 44 of 44 structure(s) from Generated Molecules
44 molecules have been found for the current spectrum query.

Most Probable Structure

Following structure has been placed to the first position after spectra calculation

Structure Elucidation Report for "SE_Lumlac_aftercalculation" (Page 2)

Carbon Assignment

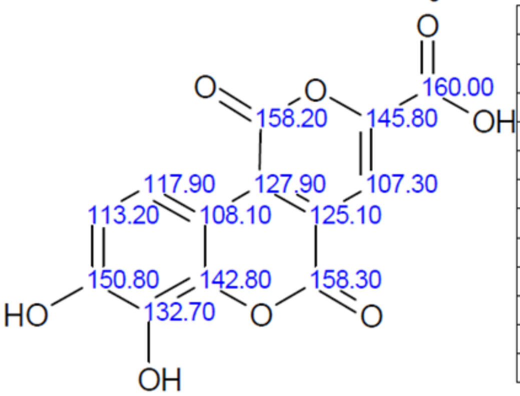

#	N	Shift (ppm)	Atoms	XHn
1	1	107.300	1	CH(d)
2	4	108.100	1	C(s)
3	2	113.200	1	CH(d)
4	3	117.900	1	CH(d)
5	5	125.100	1	C(s)
6	6	127.900	1	C(s)
7	7	132.700	1	C(s)
8	8	142.800	1	C(s)
9	9	145.800	1	C(s)
10	10	150.800	1	C(s)
11	11	158.200	1	C(s)
12	12	158.300	1	C(s)

Proton Assignment

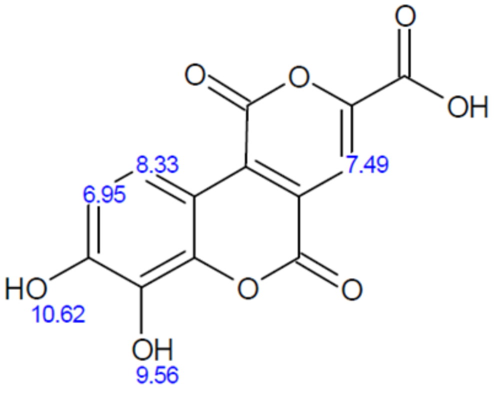

#	N	Mark	Shift (ppm)	Protons
1	2		6.950	1
2	1		7.490	1
3	3		8.330	1
4	14		9.560	1

Figure S8-1. Part of ACD-SE report

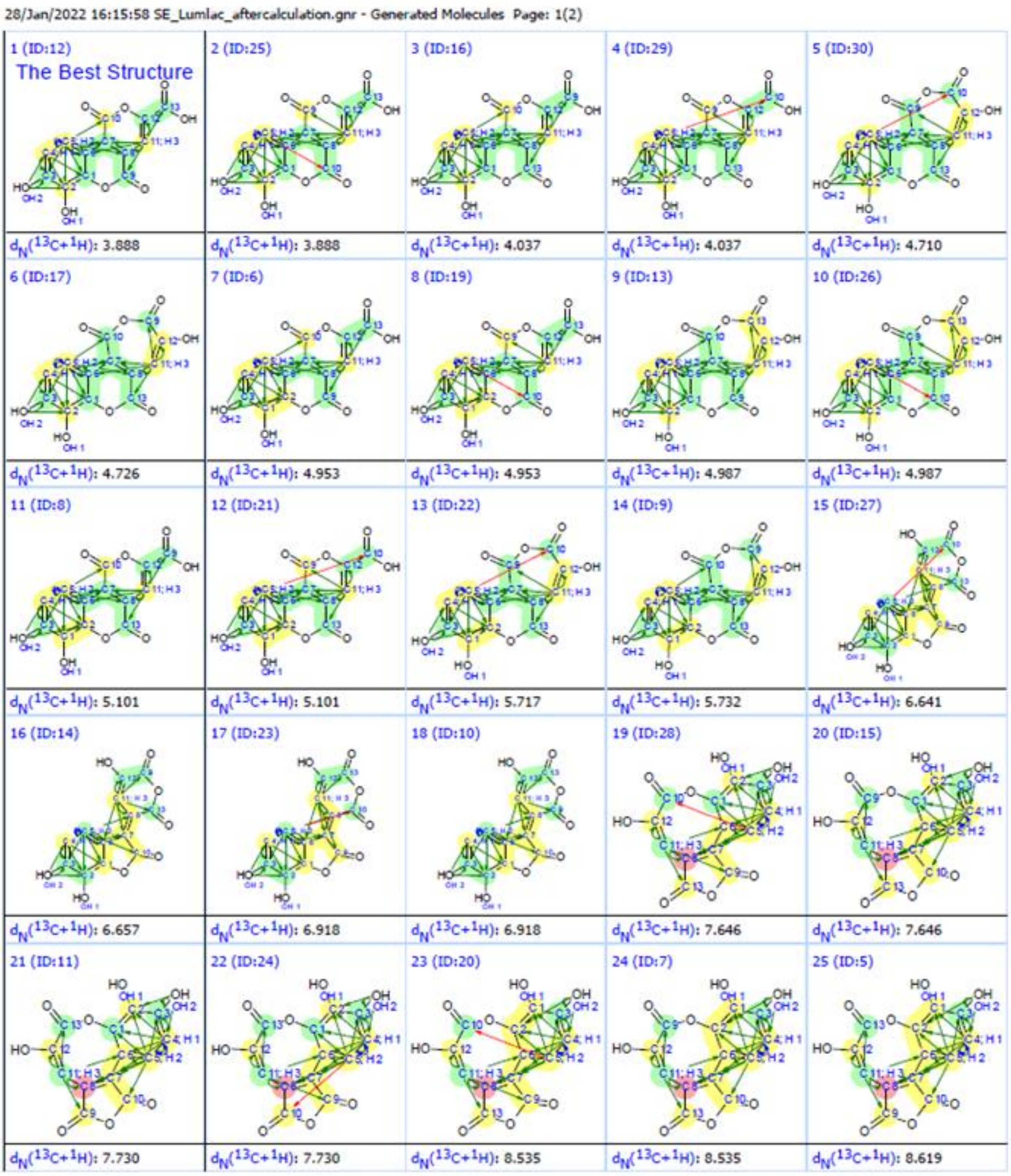

Figure S8-2. Overview of structures generated by ACD-SE

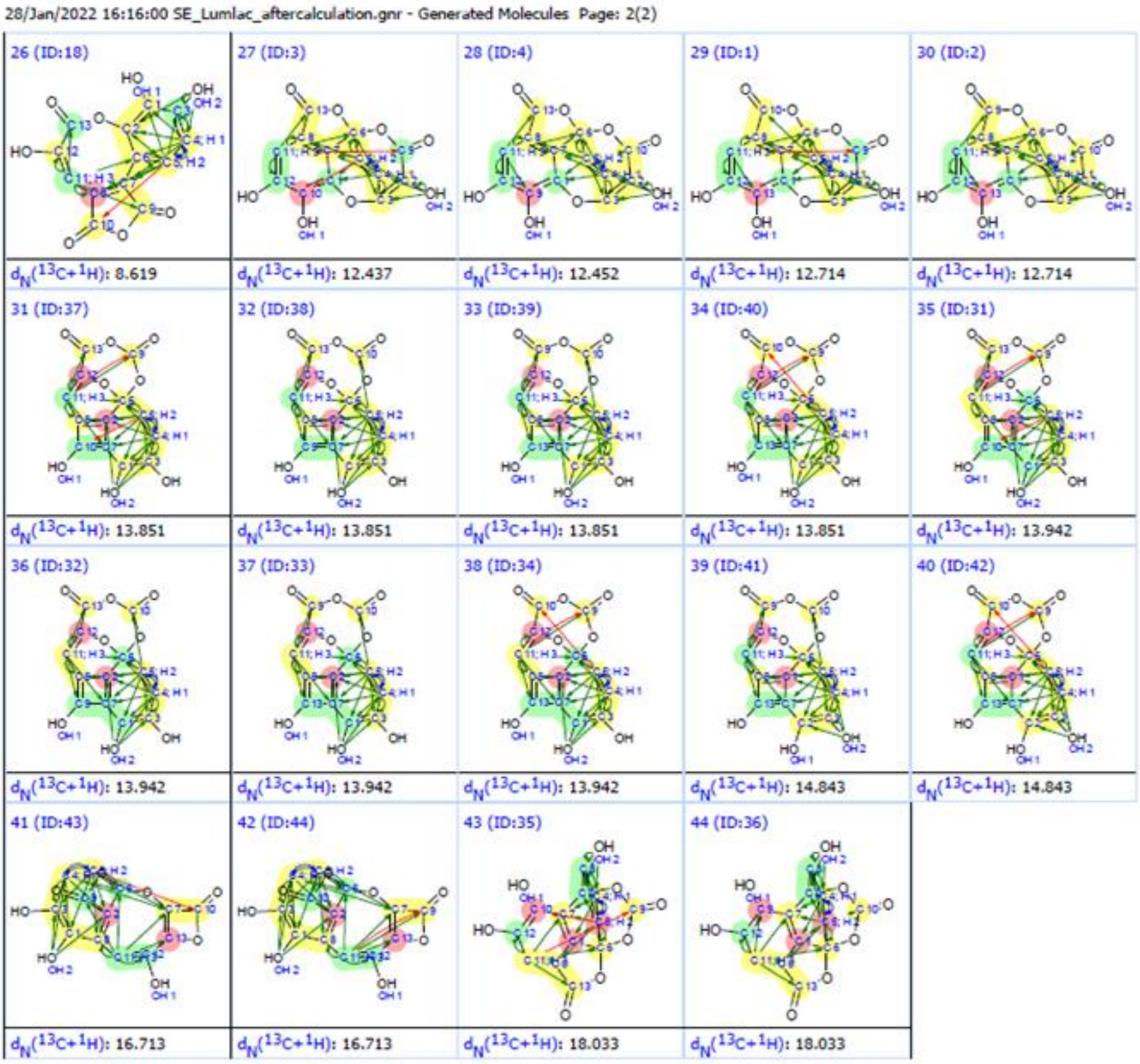

Figure S8-2, continued. Overview of structures generated by ACD-SE

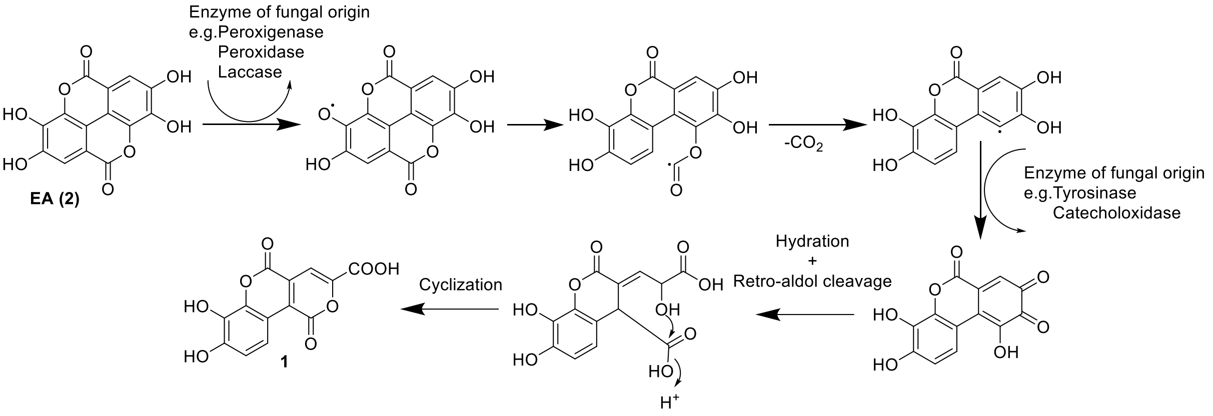

Scheme S2-1. A suggested pathway for the biosynthesis of lumnitzeralactone (**1**), including a radical induced decarboxylation step (dark pathway)

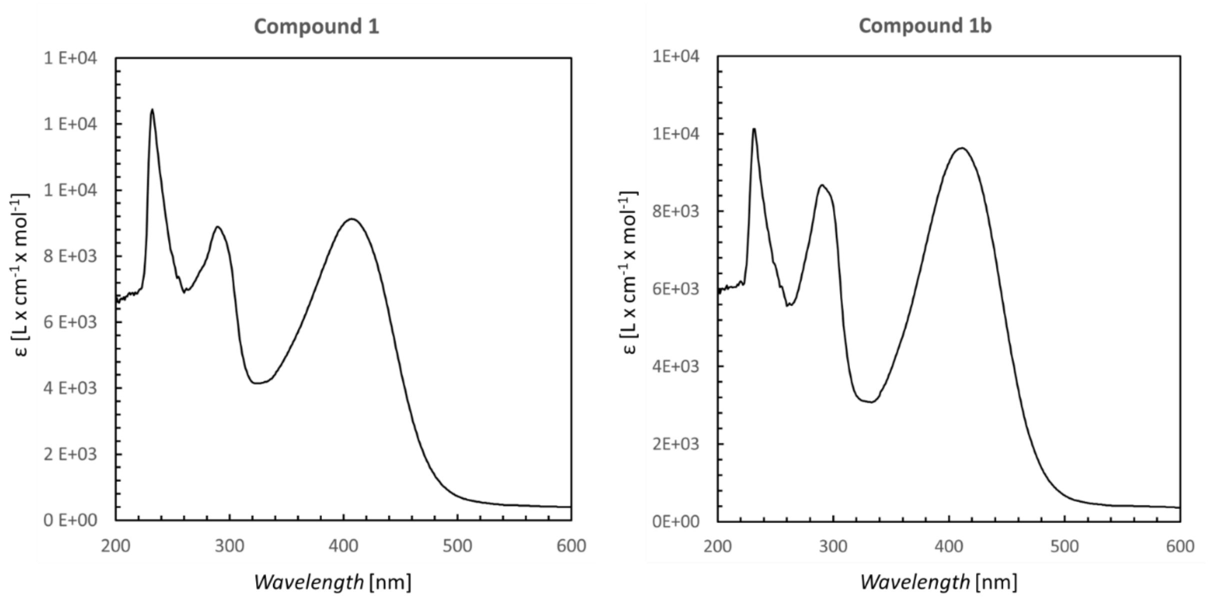

Figure S9-1. UV spectra of isolated compound **1** and synthetic **1b**

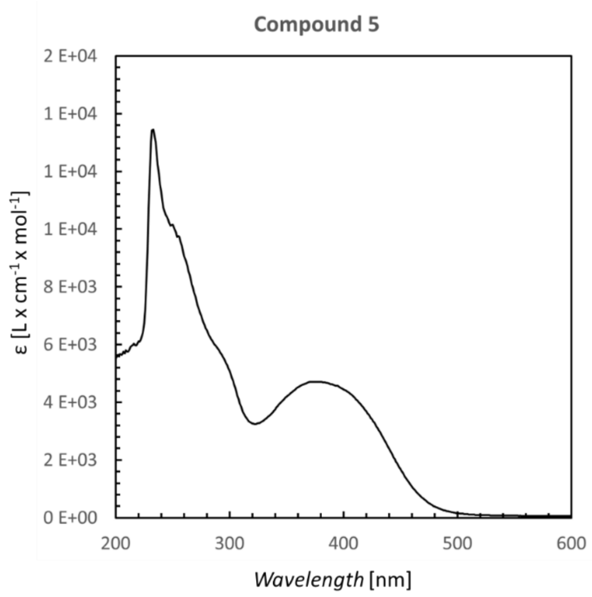

Figure S9-2. UV spectrum of compound **5**

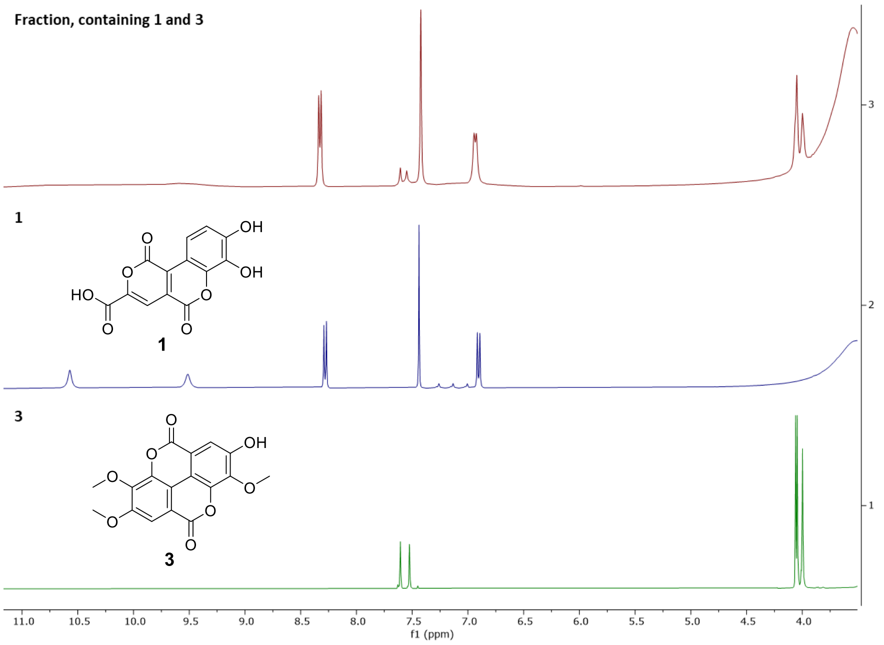

Figure S10-1. ^{1}H NMR spectrum of the antibacterial fraction containing **1** and **3** compared to the isolated compounds **1** and **3** in DMSO-*d6*, 400 MHz.

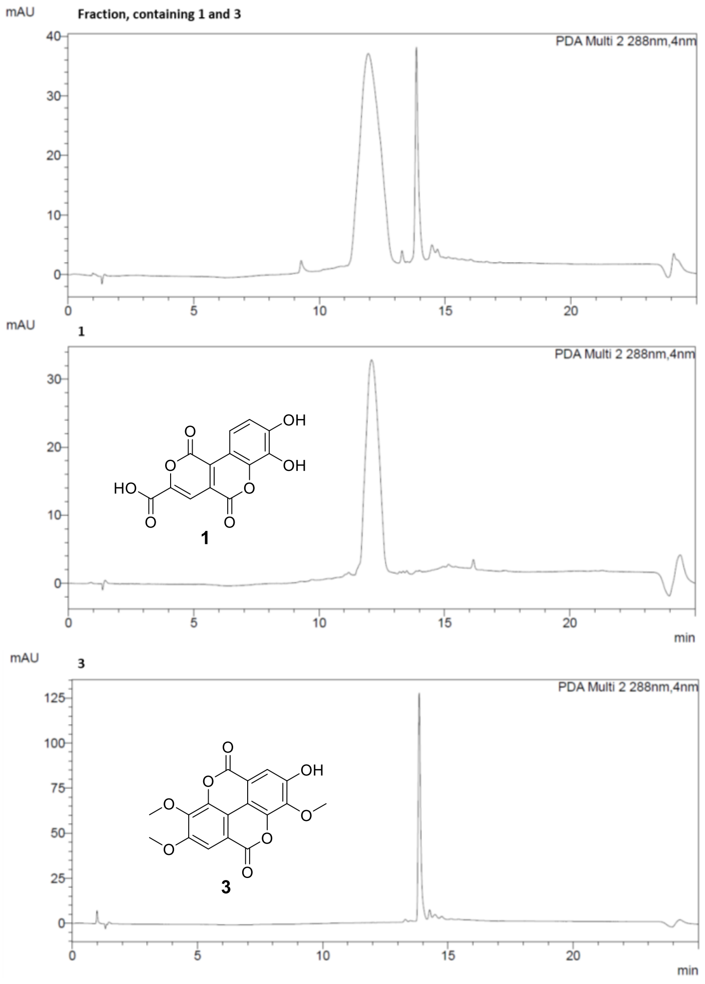

Figure S10-2. HPLC chromatogram of the antibacterial fraction containing **1** and **3** compared to the isolated compounds **1** and **3** (YMC-Triart C18; water (A)/methanol (B) gradient: 0-5, 5% B; 2-12 min, 5-100% B; 12-20 min, 100% B isocratic, flow rate of 1.5 mL/min).
